# Temperate freshwater soundscapes: A cacophony of undescribed biological sounds now threatened by anthropogenic noise

**DOI:** 10.1371/journal.pone.0221842

**Published:** 2020-03-18

**Authors:** Rodney A. Rountree, Francis Juanes, Marta Bolgan

**Affiliations:** 1 The Fish Listener, East Falmouth, Massachusetts, United States of America; 2 Biology Department, University of Victoria, Victoria, British Columbia, Canada; 3 Laboratoire de Morphologie Fonctionnelle et Evolutive, Institut de Chimie, Université de Liège, Liège, Belgium; University of Windsor, CANADA

## Abstract

The soundscape composition of temperate freshwater habitats is poorly understood. Our goal was to document the occurrence of biological and anthropogenic sounds in freshwater habitats over a large (46,000 km^2^) area along the geographic corridors of five major river systems in North America (Connecticut, Kennebec, Merrimack, Presumpscot, and Saco). The underwater soundscape was sampled in 19 lakes, 17 ponds, 20 rivers and 20 streams, brooks and creeks that were grouped into broad categories (brook/creek, pond/lake, and river). Over 7,000 sounds were measured from 2,750 minutes of recording in 173 locations over a five-week period in the spring of 2008. Sounds were classified into major anthropophony (airplane, boat, traffic, train and other noise) and biophony (fish air movement, also known as air passage, other fish, insect-like, bird, and other biological) categories. The three most significant findings in this study are: 1) freshwater habitats in the New England region of North America contain a diverse array of unidentified biological sounds; 2) fish air movement sounds constitute a previously unrecognized important component of the freshwater soundscape, occurring at more locations (39%) and in equal abundance than other fish sounds; and 3) anthropogenic noises dominate the soundscape accounting for 92% of the soundscape by relative percent time. The high potential for negative impacts of the anthropophony on freshwater soundscapes is suggested by the spectral and temporal overlap of the anthropophony with the biophony, the higher received sound levels of the anthropophony relative to the biophony, and observations of a significant decline in the occurrence, number, percent time, and diversity of the biophony among locations with higher ambient received levels. Our poor understanding of the biophony of freshwater ecosystems, together with an apparent high temporal exposure to anthropogenic noise across all habitats, suggest a critical need for studies aimed at identification of biophonic sound sources and assessment of potential threats from anthropogenic noises.

## Introduction

Fish sounds were first scientifically studied in North America by Abbot [1] who in 1877 lamented that “the little fishes of our inland brooks and more pretentious denizens of our rivers are looked upon as voiceless creatures…” and concluded that “certain sounds made by these fishes are really vocal efforts….”. Almost 100 years later Stober [2] was the first to describe the underwater noise spectra in relation to freshwater fish sounds in any freshwater habitat in an effort to determine if the sound of streams entering a lake could be used as a homing cue for cutthroat trout. He also pointed out the critical lack of data on the ambient noise and fish sounds in freshwater systems. Fortunately, after long neglect there has been a recent surge in interest in the potential impacts of anthropogenic noise on freshwater ecosystems [see reviews in 3–9], but efforts to understand such impacts are hampered by the paucity of data on the natural soundscape composition [9]. The need for research on the sound production of freshwater fishes was highlighted in our recent review of the literature, which found that sounds have been reported in only 87 species in North America and Europe, but detailed descriptions of sound characteristics are known for only 30 species [9].

Acoustic investigations of freshwater soundscapes can be grouped (S1 Table and citations therein) into those that seek to characterize the ambient noise [2, 10–20], those that focus on the biophony (without reporting on anthropophony) [21–27], those that focus on noise impacts on fishes and other organisms (see reviews in [8, 28–30]), and those that include some information on both the biophony and anthropophony [31–43]. Most of the latter studies focus on sound levels and do not provide information on the relative contribution of both the biophony and anthropophony to the soundscape in terms of percent occurrence, number of sounds, percent of time occupied, or diversity (except [31,32, 35,36, 40] S1 Table). No freshwater study provides data on the rate of temporal overlap between anthropomorphic and biophonic sounds (i.e., how often do fish sounds overlap with anthropogenic sounds?). Descriptive studies that characterize the soundscape of freshwater habitat including the relative contribution of both anthropogenic and biophonic sounds to the soundscape composition are needed. Furthermore, only a few studies incorporate some level of real-time acoustic monitoring into the sampling design (S1 Table, [2, 23, 27, 31]).

Previous studies of the biophonic component of freshwater habitats in the New England region of North America include species-specific studies [23, 44,45] and a soundscape survey of the Hudson River [31]. Martin and Popper [42] conducted a survey of the noise levels in the vicinity of the Tappan Zee Bridge in the Hudson River, but did not quantify the occurrence of biophonic sounds.

The present study is part of a series of studies seeking to document the soundscape composition of habitats in New England. In a pilot study focusing on fish sounds in the Hudson River a high diversity of known and unknown biological sounds was discovered [31]. At a location in New York City, arguably one of the world’s most impacted locations, a high diversity of mostly unknown biological sounds was heard at night likely resulting from a combination of increasing nocturnal sound production and decreasing masking from boat noise [31]. The value of reporting information on unknown biological sound occurrence was emphasized after the discovery that one of the unknown sounds in the Hudson River was produced by the invasive freshwater drum, *Aplodinotus grunniens* [44].

Following the Hudson River study, this study was conducted to document the soundscape of a wide range of habitats within the New England region. Because real-time monitoring was conducted during all recording, it was possible to notice that some of the most common sounds appeared to be of the little understood air passage type. It was, therefore, necessary to conduct a series of surveys over the next ten years to identify some of the air passage sounds and to confirm that they could confidently be attributed to fish [45]. Air passage sounds, which hereafter are referred to as “air movement” sounds, are produced by internal movements of gas to and from the gas bladder, or release of gas through the mouth, gill, or anal vent in physostomous fishes [45]. The best-known air movement sound is the “fast repetitive tick” (FRT) type which consists of a broadband high-frequency burst followed by a train of repeating ticks [45]. This new understanding of air movement sounds occurring in freshwater fish allowed us to reprocess recordings collected in the 2008 study in order to classify sounds into broad biological sound types.

The primary goals of this study were to document the occurrence of biological sounds in a large variety of freshwater habitats over a large geographic area and to determine the relative contribution of broad categories of both biological and anthropogenic sounds to the overall aquatic soundscape composition.

## Materials and methods

### Ethics statement

No ethics statement or permit were required for this non-invasive observational study.

### Study area

**Fig 1:**
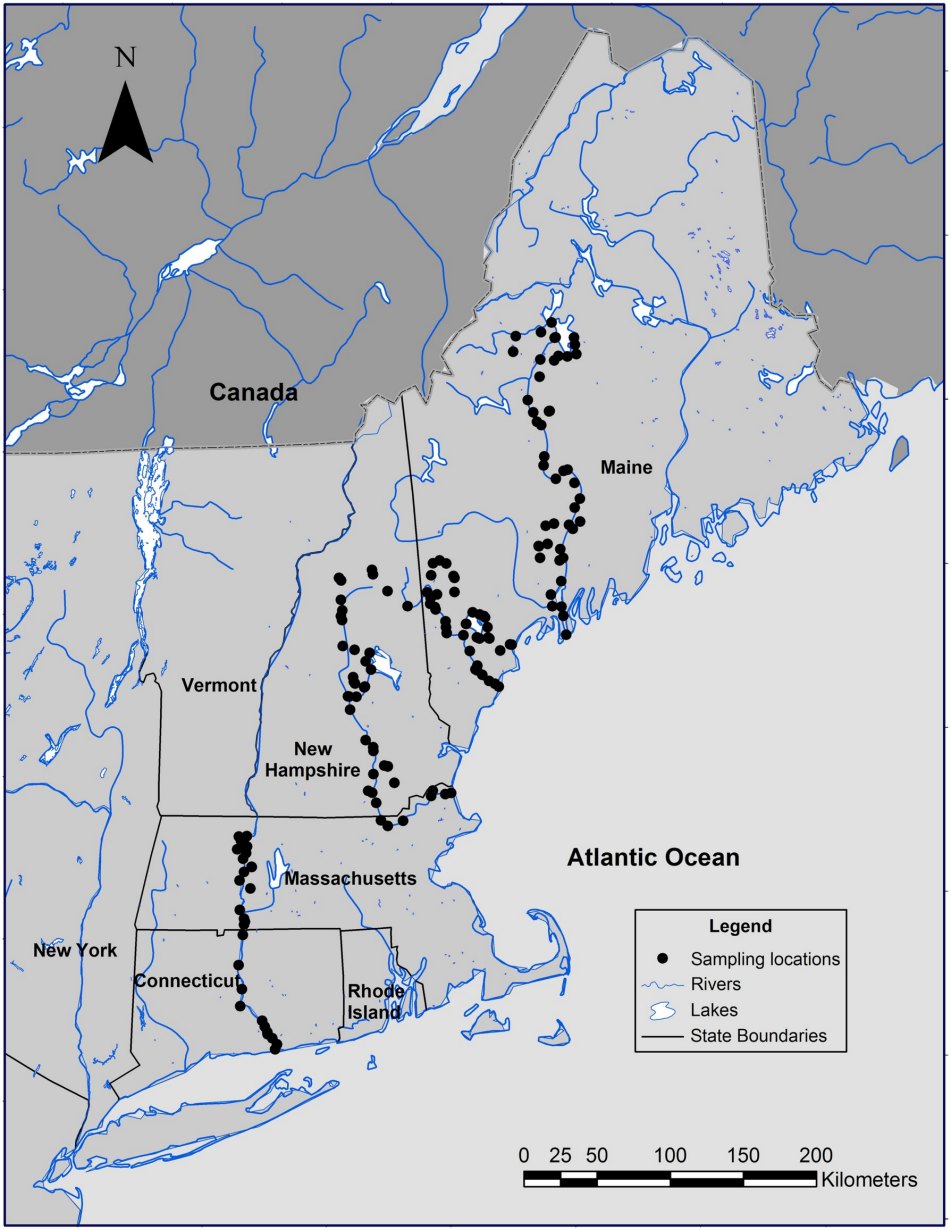
Study area. Locations where sounds were recorded between 30 April and 30 May 2008 (Location coordinates and other metadata are provided in S1 Data set).

The underwater soundscape of freshwater habitats was sampled in a roving survey within a 46,000 km^2^ region along the corridors of five major rivers in the New England region of North America over a five-week period from 30 April to 29 May 2008 (Fig 1, all location and metadata are provided in S1 Data set). River corridors surveyed included the 653 km Connecticut River (30 April to 3 May, N = 32), 188 km Merrimack River (12–16 May, N = 43), 270 km Kennebec River (17–23 May, N = 53), 219 km Saco River (26–28 May, N = 31) and 42 km Presumpscot River (28–29 May, N = 14). Within these five major rivers, sound recordings were made from shore within the main stems of each major river from its origin in the mountains or major lakes to its outlet to the sea, except for the Connecticut River where only the lower 200 km were surveyed (N = 20, 18, 20, 16, and 5 locations for the Connecticut, Merrimack, Kennebec, Saco, and Presumpscot, respectively). In addition, sound recordings were made from a large variety of other habitats including 15 river tributaries (N = 20 locations), 19 lakes (N = 32), 17 ponds (N = 18), and 20 streams, brooks and creeks (N = 24). Access to the water was often difficult and usually required sampling within 500 m of a bridge (45%) or dam (12%), and sometimes involved scrambling down steep ravines, over rocks, or short hikes through woodlands (S1 Data set). All locations were photo-documented. Although both lotic and lentic habitats sampled represent a continuum, for comparison purposes, small brooks and streams were combined with small sluggish rivers or creeks into a “brook/creek” habitat category, small ponds and large lakes were combined into a “pond/lake” category, and tributary and main stem river sites were combined into a “river” category (S1 Fig, S1 Data set).

A total of 173 sites were successfully sampled during this survey (S2 Table). Sampling was avoided during poor weather (high winds and/or rain). Sampling occurred during the day between 0930 and 1900 (N = 148), and at night between 1900 and 2300 h (N = 25). Recording durations were from 1 to 49 minutes (mean = 12.5 min) during day and 3 to 119 minutes (mean = 35.5 min) during night sampling (S2 Table). Night recordings were longer on average than day recordings because we expected increased biological activity around sunset based on previous experience [13, 31–33, 44,45].

### Acoustic data

Acoustic data were captured from a low noise (nominal sensitivity -165 dB, re 1 μPa), broadband (flat response deviating less than ± 1 dB from 16–44,000 Hz), cylindrical hydrophone with 30 m of cable (model C54XRS, Cetacean Research Technology (CRT), Seattle, Washington) at 48kHz (24 bit) with a MOTU Ultralite, bus powered firewire audio interface to a laptop computer using SpectraPro332 Professional Sound Analysis software (Sound Technology, Inc.). The entire system including the hydrophone, audio interface, laptop, and SpectraPro 322 software was provided by CRT with preconfigured calibration settings for two fixed gains (zero and half full-scale on the audio-interface). All recordings were made with the same equipment and the same two fixed calibrated gain settings. Data were automatically converted to sound pressure level (SPL dB re 1 μPa) by SpectroPro322 in real-time during field recording. Sound from either a dubbing microphone or a second hydrophone was captured to a second channel in the recordings. When used, the second hydrophone was an uncalibrated, variable (dial) gain Aquarian model (Aquarian Audio Products, Anacortes, WA, USA). All recordings were monitored aurally and visually in real-time with Spectropro322 displaying the spectrogram and waveform in SPL dB re 1 μPa. Written and oral notes on sounds together with potential environmental, anthropogenic and biological sound sources were also recorded throughout each recording. Additional post-processing of acoustic signals was conducted by listening to all recordings in their entirety while simultaneously viewing the spectrogram (1024 FFT, Hanning window, 50% overlap) and waveform with Raven Pro 1.5 acoustic software (Bioacoustics Research Program 2014).

Most anthropogenic sounds (anthropophony) were positively identified in the field, including airplane, boat, traffic (bridge crossing or road), train and other noise. Boat sounds were divided into sounds of running boats, boats at idle, and other boat sounds (engine cranking, pumps, etc.). Fishing sounds were those made by recreational fly and spin-cast fishers and included lines hitting the water surface and sinking, lure retrieval, weights dragging on the bottom, etc. Other noise included miscellaneous construction sounds (e.g., hammer tapping) lawn mowers, fish/depth finders, etc.

Biological sounds were classified into broad categories on the basis of previous knowledge of freshwater biophony [13, 31–33, 44,45] and the real-time field observations. Biological sounds were classified as fish, insect-like, surface, bird and other biological. Fish sounds were subdivided into air movement sounds and other fish sounds. Air movement sounds were further divided into FRT and other air movement sounds. An example of an air movement sound associated with a fish jump is provided in S1 Audio (including voice notes), more detailed descriptions and examples of these types of sounds are presented in our companion paper [45]. Other fish sounds included more conventionally recognized fish sounds such as drumming and stridulation sounds which can be pulsed or tonal. Bird sounds were recorded underwater from sounds arising above water (as determined during real-time monitoring), we include them in the underwater biophony despite their external origin because they are in fact part of the aquatic soundscape in the same way that many anthropogenic sounds that arise from terrestrial and aerial sources are considered part of underwater soundscapes. Because we have previously observed that the sounds made by fishes when they jump or gulp air at the surface can be useful identification markers, and may potentially have inter- or intra-biological functions [45], we include them in a “Surface” sound category. Other biological sounds included beaver (tail slaps) and seal sounds, as well as unknown snapping sounds. Unknown sounds deemed to be biological based on their frequency and temporal pattern but which could not confidently be placed within one of the biological categories were also placed in the other biological sound category. The unclassified sound category included bubble-like and gurgle sounds because a type of air movement sound labeled “gurgle” described for salmonids cannot yet be reliable distinguished from natural sounds of gas release from the sediment [45].

Aggregate sound categories included air movement (FRT + other air movement), fish (air movement + other fish), biophony (fish + insect-like + bird + surface + other biological), boat (running boat + idle boat + other boat), anthropophony (airplane + boat + fishing + traffic + train + other noise), and unclassified (gurgle + bubble-like + unknown).

Within each recording, unique biological sound types were annotated and subjectively labeled (e.g., FRT-A, FRT-B) and the number of unique biological sound types counted (i.e., biological sound diversity per recording). However, while biological sound categories were consistent across all recordings, individual sound types within each biological category were not, due to the high variability of sound characteristics, and diversity of habitats sampled, so total biological sound diversity could not be determined. Many sounds were unique to specific locations. For example, a sound labeled a “bark” and placed in the other fish category in one recording might have different acoustic characteristics from a “bark” in another recording, making it difficult to determine if the observed differences were due to variability or different sources. However, in that case the sound could still be confidently placed in the “other fish” category. In addition, two 5 s clips from each recording were annotated to represent the background ambient noise, herein defined as the background sound when no individually recognizable biological or anthropogenic sounds were observed [*sensu* 12]. Ambient noise includes relatively constant acoustic energy, but no individually recognizable sound, from the geophony, biophony and anthropophony.

Acoustic measurements of all sounds were made in Raven of selected parameters including duration, peak frequency and frequency bandwidth [46], and data pooled within the appropriate sound type category. The “percent frequency of occurrence” of sounds among recordings was determined as the number of recordings containing a sound type, divided by the total number of recordings and should not be confused with acoustic frequency parameters. To partially account for bias due to recording length, accumulation curves were calculated for each location as the number of sound types plotted against time elapsed from the beginning of each recording. Because the accumulation rates were highly variable among locations and did not exhibit an asymptote, it was necessary to use only portions of recordings that were comparable among locations to compute diversity. Thus, a standardized diversity was calculated as the number of sound types observed up to 348 s after dropping recordings with less than 174 s total duration (N = 165). Percent occurrence calculated from all recordings were similarly compared against a standardized recording length, but because trends and statistical tests were the virtually the same, we report percent of occurrence based on all recordings herein.

Sound rate was calculated as the number of sounds per minute in each recording for each sound category. Percent recording time for each category was calculated by summing the durations of all individual sounds in the category in a recording and dividing by the recording duration. In order to examine time-of-day trends, the data were reconfigured to count the number of sounds of selected types in portions of recordings that corresponded to specific hours of the day. The hourly counts were then pooled over all recordings to obtain time-of-day curves. Interpretation of the resulting data is cautioned because estimates for some hours are based on a small number of observations recorded from highly variable locations and dates.

Temporal overlap among biological and anthropogenic sounds was determined by counting the number of sounds that overlapped completely, or partially, in time (i.e., that occurred at the same time in the recording).

Received sound pressure levels (RSPL) as root mean square (RMS [47,48]) were automatically calculated in SpectraPro332 over the full 24 kHz bandwidth for each ambient clip and used to assign each recording to an ambient noise level category ranging from low-to-high in 5 dB steps (90–95, 96–100, 101–105, and >105 dB RMS RSPL re 1 μPA). Although these sound level categories should not be construed to be estimates of the true location noise levels, which likely vary widely over time and space, they do represent the received background noise levels affecting the soundscape during the recording time. Sound levels were not estimated in some recordings due to mechanical noise on the calibrated hydrophone, strong flow, cable strumming, or other factors. Many locations (28%) apparently exhibited electromagnetic field (EMF) levels high enough to introduce EMF noise into the recording and were excluded. Power spectral density (PSD, Hanning, FFT 4096, 50% overlap, frequency resolution 11.7, PSD dB re 1 μPa^2^Hz^-1^) data were automatically calculated and exported from Spectrapro322 for each identified sound over its frequency bandwidth, and for each ambient noise clip over the full recorded bandwidth (Nyquist frequency = 24 kHz). Average PSD spectra of each sound category, including ambient, were then calculated from a subsample (S3 Table) of representative clips.

Because PSD spectra of sound types contain energy from the background ambient noise, and because sound categories occurred in various sized subsets of the recordings (i.e., some categories were relatively rare, while others were ubiquitous), a comparison of the mean PSD spectra of individual sound categories with mean ambient noise PSD can be misleading. To address this bias, the sound levels of the ambient noise were subtracted from the sound levels of selected sound categories based only on the locations in which they actually occurred. For example, after linearizing, the spectra of the ambient noise were subtracted from each FRT subsample from each of the ten locations where the FRT subsamples were selected, and then all the resulting spectra were averaged over all 23 FRT subsamples. The resulting PSD spectra provide an indication of the frequency bands over which the biophony is substantially higher than the ambient, and comparisons with the PSD spectra of the biophony with that of the anthropophony after subtraction of the ambient gives an indication of the potential for masking.

### Data analyses

Due to the wide variety of habitats sampled and lack of temporal control (data were not synoptic and of variable duration), many environmental factors that potentially influence soundscape composition such as habitat category, diel period, river position and river system were statistically confounded preventing detailed statistical comparison of their effects. However, we cautiously compared selected factors based on various subsamples of the data pooled within broad treatment categories in an exploratory examination of their potential to influence the soundscape composition. The exploratory nature of these comparisons is emphasized given the “snap-shot” nature of the data collection and lack of control of environmental variables such as time-of-day, temperature, season (although all data were collected over a five-week period), amount of human development (ranging from remote wilderness to heavily populated urban areas), turbidity, water depth, and propagation distance. Because strong diel differences were expected, and night sampling was limited, all soundscape measurements (percent frequency, sound rate, and percent time) are reported separately for day and night recordings. Factors examined for possible influence on the soundscape composition included diel period, habitat type, position along the river gradient (examined separately for each river), river region (non-tidal and tidal reaches pooled over all rivers for day samples) and ambient noise level category. Frequency of occurrence of sound types among diel, habitat type and ambient noise level category groups were tested with a Chi-Square test in one-way contingency tables. A one-way analysis of variance was used to test for potential single-factor effects (e.g., habitat category) sound number and time after transforming to normalize.

## Results

A total of 4,825 biological, 1623 anthropogenic noise, and 834 unclassified sounds (Table 1) were measured from 2,750 minutes of recording in 173 locations (S2 Table). Examples of some of the most common air movement sounds are described elsewhere [45]. Examples of traffic, train, running boat and other boat noises are provided in S2–S5 Figs together with their corresponding sound files (S2–S5 Audios). An example demonstrating likely masking of other fish, other air movement, and FRT sounds by traffic noise can be viewed in S2 Fig and heard in S2 Audio. These examples are provided to demonstrate the temporal variation in frequency content and relative sound level change within the sound type.

**Table 1:** Sound duration and peak frequency.

	Duration (s)					Frequency (Hz)					
Sound category	Samples	Mean	SE	Min	Max	Samples	Mean peak	SE peak	Max peak	Mean IQR-BW	SE IQR-BW
**Biophony**											
FRT	135	3.03	0.34	0.16	31.53	125	1717	102	4969	586	46
Other air movement	1636	0.19	0.01	0.02	13.29	1506	1742	28	10359	607	14
Other fish	1807	0.32	0.03	0.01	32.90	1612	726	9	2625	303	5
Insect-like	631	2.21	0.10	0.02	29.62	238	2820	121	12188	776	94
Bird	57	2.29	0.39	0.11	16.02	54	2912	195	4781	752	79
Surface	197	0.84	0.04	0.06	4.60	188	906	35	3797	414	23
Other biological	362	0.20	0.04	0.01	13.05	325	1811	87	11438	933	56
Subtotal	4825					4048					
**Anthropophony**											
Airplane	11	27.58	3.66	14.76	51.18	9	307	130	984	120	24
Fishing	74	2.51	0.57	0.16	32.20	67	1483	230	8344	1133	244
Running boat	57	174.64	18.73	15.37	733.44	45	875	126	4266	1094	333
Idle boat	14	276.66	68.96	14.19	788.62	11	435	162	1406	464	138
Other boat	127	6.97	1.30	0.06	100.62	75	1159	90	3609	523	53
Traffic	1237	7.90	0.26	0.05	132.00	1070	225	19	4594	151	6
Train	22	44.53	29.81	0.06	654.99	5	459	103	563	84	50
Other noise	81	15.70	2.58	0.01	182.91	63	847	99	2906	373	48
Subtotal	1623					1345					
**Unclassified**											
Gurgle	313	1.24	0.11	0.09	18.13	296	1108	20	3797	229	10
Bubble-like	105	2.39	0.24	0.06	17.44	88	948	29	2109	222	21
Unknown	416	1.02	0.10	0.01	18.24	334	1616	69	10172	580	44
Subtotal	834					718					
**Ambient**						159	297	34	1875	481	84
**Total**	7282					6270					

Summary statistics for sounds pooled over all samples within sound categories. SE = Standard error of the mean, Min = minimum value, Max = maximum value, peak = frequency of greatest energy, IQR-BW = interquartile bandwidth.

**Fig 2:**
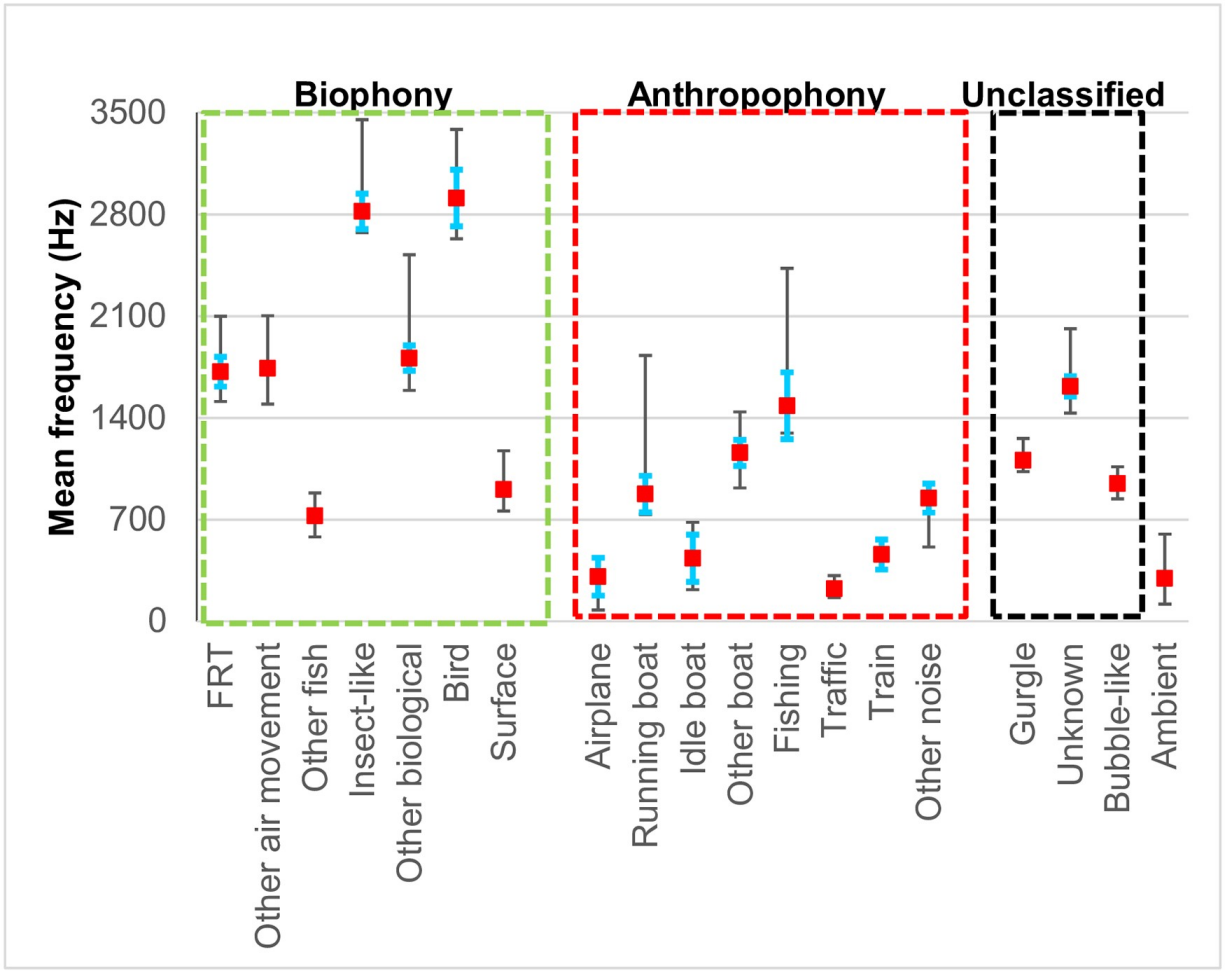
Sound duration and peak frequency. Comparison of acoustic characteristics among sound types and ambient noise. Square symbols mark the mean peak frequency, blue hats mark one standard error of the mean. The lower stem marks the mean first quartile frequency and the upper stem the mean 3rd quartile frequency (see Table 1).

In general, anthropogenic noises were 1 to 2 orders of magnitude longer in duration than biological sounds and exhibited consistent spectral overlap with them (Fig 2, Table 1, S6 Fig). FRTs, insect and bird sounds had the longest durations of the biophony averaging 2–3 s, while boat, plane and train sounds had the longest durations of the anthropophony averaging 28–277 s. Other fish sounds had the lowest peak frequency of the biophony, while insects and birds had the highest (Table 1). Traffic sounds had the lowest mean peak frequency and fishing and other boat sounds had the highest peak frequency of the anthropophony. Train sound peak frequency was inflated by whistle sounds, and otherwise would have the lowest peak frequency (S6 Fig). Peak frequency of ambient noise was below that of most biophonic sounds but overlapped strongly with traffic and other anthropogenic noises (Fig 2, Table 1, S6 Fig).

Biophony occurred in 57% and anthropophony occurred in 63% of the locations (Table 2). Other air movement sounds were the most frequently occurring (39% of locations) component of the biophony followed by other fish (30% of locations). Air movement sounds were dominated by high-frequency sounds similar to those previously described [45] for salmonids (46%), alewife (*Alosa pseudoharengus*, Clupeidae) and alewife-like sounds (27%), and white sucker-like (*Catastomus commersonii*, Catostomidae) sounds (8%). Twenty-three percent of FRT sounds were attributed to alewife while the rest were unknown. Most of the other fish sounds were unknown, but 13% were catfish-like sounds most likely produced by brown bullhead (*Ameiurus nebulosus*, Ictaluridae. [31]).

**Table 2:** Comparison of sound occurrence between diel periods.

Sound category	Day samples	Night samples	Total	Chisq P
**Biophony**				
FRT	22%	40%	24%	*
Other air movement	39%	76%	45%	***
Air movement	42%	80%	47%	***
Oher fish	30%	64%	35%	***
Fish	48%	84%	53%	***
Insect-like	14%	24%	15%	ns
Other biological	20%	44%	23%	***
Surface	14%	56%	20%	***
Bird	13%	20%	14%	ns
All biophony	53%	84%	57%	***
**Anthropophony**				
Airplane	4%	8%	5%	ns
Running boat	13%	24%	14%	ns
Idle boat	7%	0%	6%	ns
Other boat	10%	8%	10%	ns
Boat	17%	24%	18%	ns
Fishing	5%	12%	6%	ns
Traffic	37%	56%	40%	ns
Train	2%	0%	2%	ns
Other noise	10%	8%	10%	ns
All anthropophony	60%	80%	63%	ns
**Unclassified**				
Bubble-like	18%	32%	20%	ns
Gurgle	21%	48%	25%	**
Unknown	39%	72%	43%	**
All unclassified	46%	76%	50%	**
**All Sounds**	76%	92%	79%	ns
**Sample size**	148	25	173	

Percent of the stations where sound types were observed. P = probability of a significant difference in the frequency of sounds between day and night based on a Chi Square (Chisq) test in a one-way contingency table on frequency counts (ns = not significant, * = < 0.05, ** = < 0.01, *** = <0.001).

Biophony was observed in significantly more locations at night than during the day (84% vs 53%, Table 2). All components of the biophony except insect-like and bird sounds occurred in significantly more night locations than day locations. Traffic sounds were the most common component of the anthropophony occurring in 40% of the locations. No significant diel differences in occurrence among locations were observed for the anthropophony.

Other air movement (5%), FRT (7%), other fish (10%), insect-like (9%), bird (11%) and other biological (4%) sounds overlapped in time with traffic sounds. The only other noises to overlap in time with biological sounds were: airplane (0.1% and 0.4%, with other fish and insect-like), other boat (0.1% and 0.3%, with other air movement and other fish), and other noise (0.1% with other fish).

### Diel patterns

**Fig 3:**
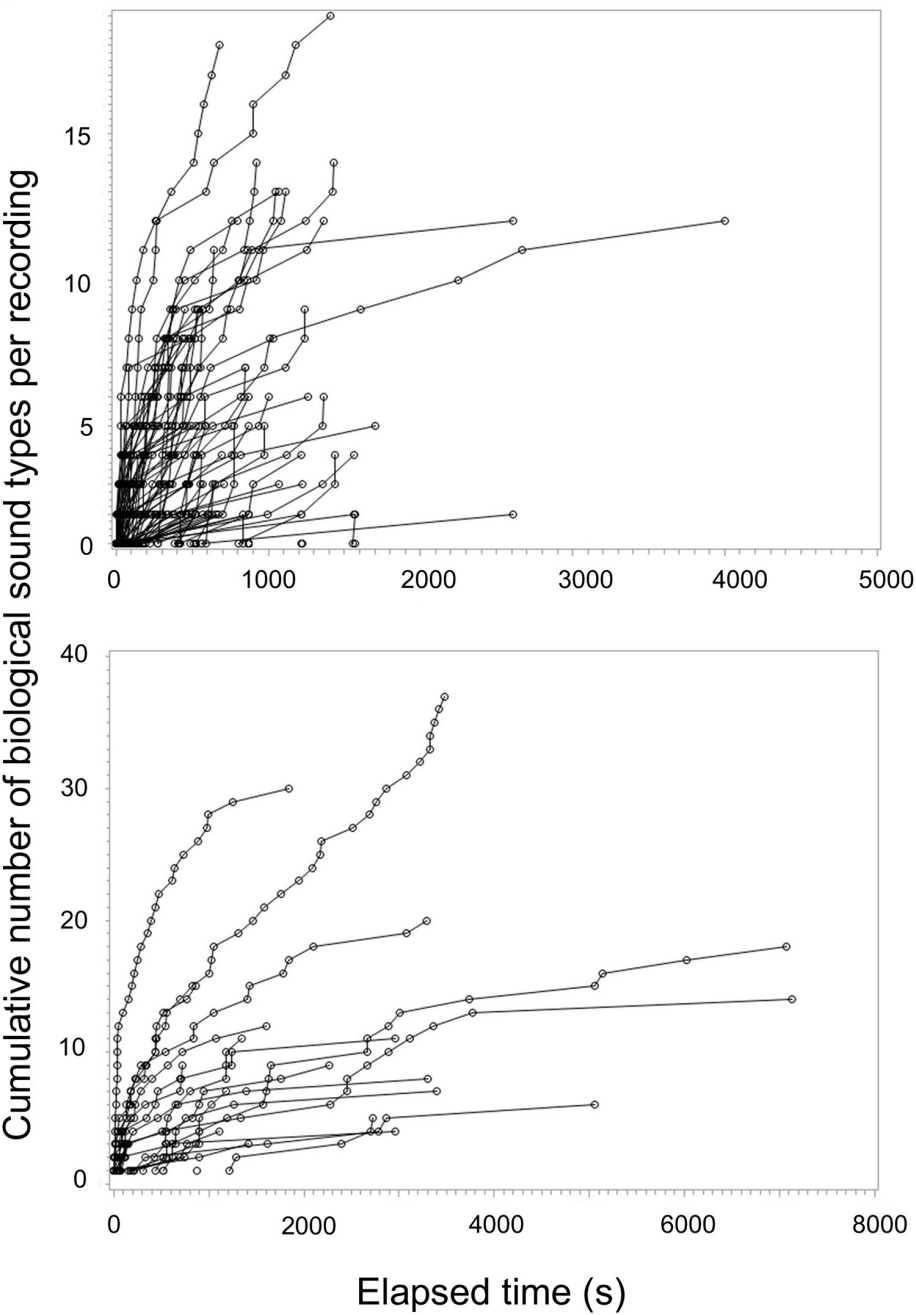
Rate of accumulation of new biophonic sound types at each location. Comparison of the number of unique types of biophony sounds for day (top) and night (bottom) plotted against the time elapsed from the beginning of the recording for each location illustrating the high variability in type number and rate of accumulation. Note the difference in the y-axis scale between day and night.

Biophony sound type accumulation curves were highly variable among locations and did not reach an asymptote (Fig 3). Total biological types per location tended to be more diverse at night averaging 9.6 types per recording (standard error (SE) = 1.8, maximum = 37) compared to 3.3 types per recording (SE = 0.3, maximum = 19) types during the day. The standardized biodiversity measure exhibited a similar diel trend, but neither the total accumulated within the first 348 s or the rate of accumulation were significantly different between day (mean = 1.8, range = 0–12, SE ± 0.2) and night (mean = 3.5, range 0–18, SE ± 0.9).

**Fig 4:**
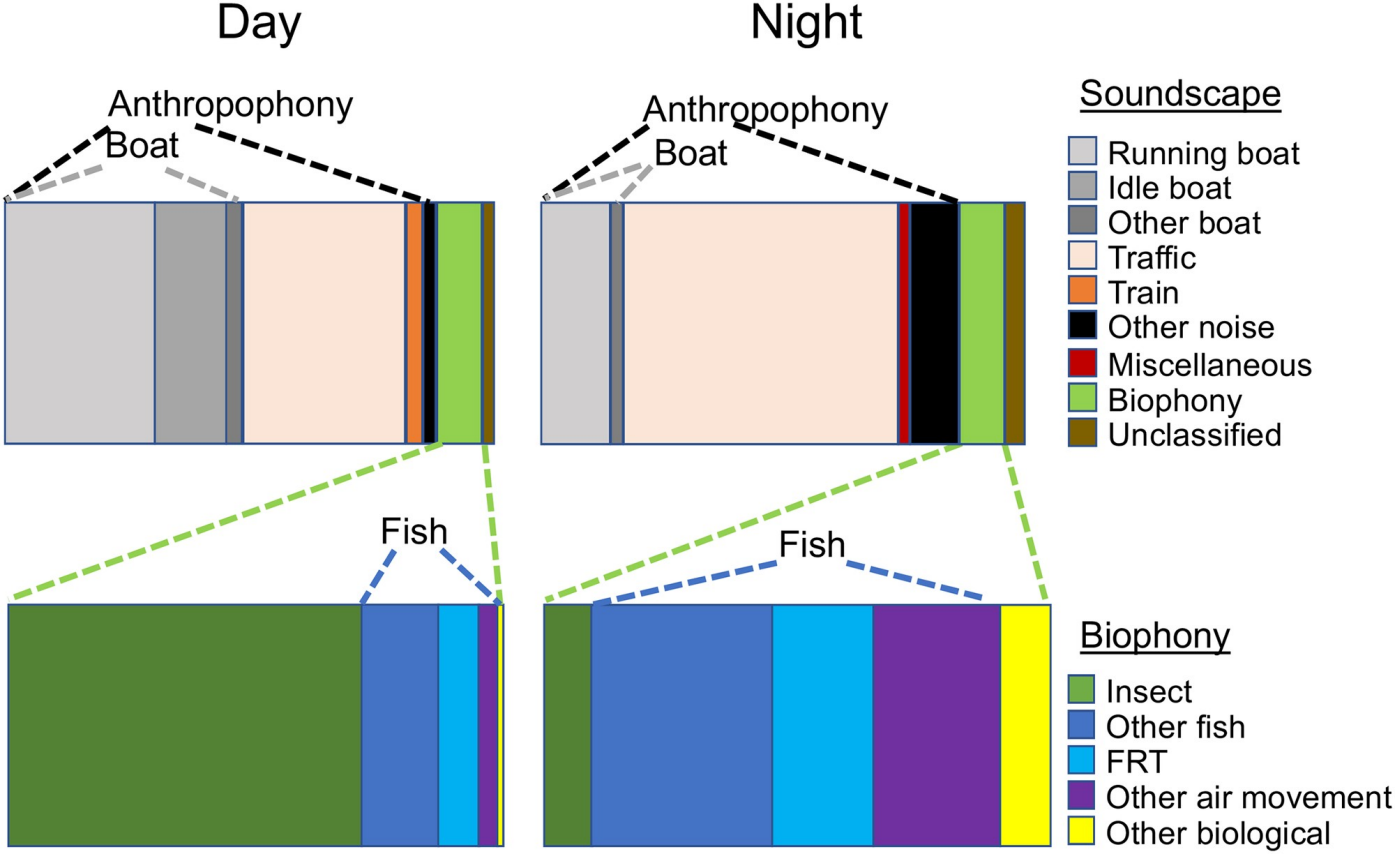
Soundscape composition. Relative contribution of the anthropophony and biophony and their major components to the aquatic soundscape during the day and night based on mean percent time of each sound type (data provided in Table 3). The composition of the biophony is shown in the expanded plots.

The biophony accounted for 66% and 67% (mean = 1.7 and 2.7 sounds/min) of the total number of sounds during the day (2.6 sounds/min) and night (4.0 sounds/min), respectively (S7 and S11 Figs, Table 3). However, the anthropophony dominated the soundscape in terms of relative percent time accounting for 92% and 88% of all sounds during the day and night, respectively (Fig 4, S7 Fig, Table 3). Unclassified sound accounted for just 9% of the total sounds by number and less than 5% of the sounds by percent time (S7 and S11 Figs, Table 3).

**Table 3:** Comparison of sounds between diel periods.

	Number per minute						Percent time					
	Day			Night			Day			Night		
Sound category	Mean	SE	Max	Mean	SE	Max	Mean	SE	Max	Mean	SE	Max
**Biophony**												
FRT	0.028	0.006	0.6	0.054	0.024	0.5	0.13	0.04	4.10	0.27	0.11	1.90
Other air movement	0.239	0.074	10.2	0.940	0.350	7.9	0.06	0.01	1.30	0.34	0.12	2.10
Air movement	0.267	0.075	10.3	0.994	0.370	8.4	0.18	0.04	4.17	0.58	0.20	4.01
Other fish	0.585	0.201	22.3	1.316	0.790	18.9	0.24	0.08	7.50	0.48	0.22	4.90
Fish	0.852	0.221	22.3	2.310	0.913	20.2	0.44	0.10	7.50	1.09	0.33	6.30
Insect-like	0.759	0.597	88.0	0.032	0.020	0.5	1.12	0.59	76.80	0.13	0.10	2.30
Other biological	0.076	0.019	1.5	0.198	0.064	1.1	0.02	0.01	0.40	0.14	0.09	2.10
Surface	0.029	0.010	0.9	0.111	0.042	1.0	0.04	0.01	1.38	0.14	0.08	2.04
Birds	0.015	0.004	0.4	0.017	0.008	0.1	0.04	0.02	1.76	0.07	0.05	1.08
All biophony	1.730	0.647	89.6	2.668	0.940	20.5	1.57	0.57	77.02	1.51	0.47	9.39
**Anthropophony**												
Airplane	0.003	0.001	0.1	0.005	0.003	0.1	0.17	0.09	9.80	0.22	0.16	3.20
Running boat	0.020	0.005	0.3	0.009	0.004	0.1	5.30	1.37	92.40	2.06	0.92	16.50
Idle boat	0.005	0.002	0.1	0.000	0.000	0.0	2.52	0.99	100.00	0.00	0.00	0.00
Other boat	0.092	0.057	8.2	0.017	0.016	0.4	0.57	0.34	46.60	0.36	0.35	8.50
Boat	0.118	0.058	8.2	0.026	0.018	0.5	8.40	1.95	100.00	2.42	1.07	16.50
Fishing	0.009	0.004	0.4	0.045	0.025	0.5	0.04	0.02	2.10	0.11	0.07	1.70
Traffic	0.453	0.087	7.7	0.824	0.482	12.5	5.77	1.05	69.80	8.22	2.89	54.80
Train	0.008	0.005	0.6	0.000	0.000	0.0	0.56	0.41	54.90	0.00	0.00	0.00
Other noise	0.025	0.009	0.9	0.033	0.026	0.6	0.21	0.18	26.00	1.44	1.14	26.50
All anthropophony	0.644	0.106	9.2	0.964	0.490	12.4	15.23	2.17	100.00	12.55	3.54	65.50
**Unclassified**												
Bubbles	0.034	0.008	0.6	0.031	0.014	0.3	0.12	0.03	2.13	0.16	0.09	1.93
Gurgle	0.045	0.009	0.7	0.199	0.104	2.5	0.10	0.03	2.42	0.34	0.18	4.10
Unknown	0.163	0.031	2.7	0.142	0.033	0.6	0.26	0.06	5.03	0.24	0.06	1.10
All unclassified	0.242	0.035	2.8	0.371	0.127	3.0	0.47	0.08	5.03	0.71	0.22	4.92
All sounds	2.616	0.660	89.6	4.002	1.166	25.4	16.55	2.12	100.00	14.26	3.58	66.02
**Sample size**	148			25			141			24		

Mean number per minute and percent of recording time per minute for all locations of selected sound categories for day and night sampling. SE = standard error of the mean, Max = maximum value.

Although the relative contribution of biophony and anthropophony by both number and percent time were similar between day and night, the composition of the sounds changed (Fig 4, S7 Fig, Table 3). Insect sounds dominated the biophony by day, while total fish sounds dominated by night. Air movement fish sounds and other fish sounds contributed about equally to total fish sounds, though other fish sounds were more numerous during the day, while the longer duration air movement sounds accounted for more of the percent time at night. Traffic sound was the most numerous component of the anthropophony during both day and night but the long-duration boat sounds contributed more to the percent time during the day. Traffic sound accounted for more than half of the recorded sound during the night based on mean percent time per location. Hourly trends for sound rates of the biophony reflect a similar diel pattern (S8 Fig). Insect-like sounds peaked at 87 sound/h in the early afternoon, air movement sounds peaked at 43 sound/h in the early evening, while other fish sounds peaked at 18 sounds/h in the afternoon and 32 sounds/h in the evening (S8 Fig).

### Habitat patterns

There was no significant difference in the frequency of occurrence of biophonic categories among habitat types during the day (S4 Table). At night, insect sounds occurred more frequently in the brook/creek habitat locations (however, the brook/creek sample size was low N = 2), while other biological and bird sounds were absent from the pond/lake habitat locations. Traffic sounds were the most widespread noise and were significantly more frequently occurring in brook/creek habitat locations during the day. In contrast, boat sounds were absent from brook/creek locations (S4 Table). No significant differences in the frequency of occurrence of anthropophonic sounds were observed among the habitat categories at night (S4 Table).

Insect and other fish sounds dominated the biophony by percent time in all habitats during the day, but air movement sounds dominated pond/lake and river habitats at night (S9 Fig, S5 Table). Other fish dominated brook/creek habitat at night but the sample size was low (N = 2 locations). Traffic sounds dominated soundscape percent time during both day and night in brook/creek habitat, while boat sounds dominated other habitats (S9 Fig, S5 Table).

### River gradient pattern

There were no consistent trends among rivers in biological or noise sounds in main-stem river habitats moving along the river gradient from headwaters to mouth, although the highest elevation locations tended to have little or no biological sounds (S1 Data set online). When day data from all rivers were pooled after grouping locations into non-tidal (N = 46) and tidal regions (N = 20), all boat noise categories were significantly more frequent in tidal regions (S6 Table). Similar trends were observed for sound rate and percent time (S10 Fig, S6 Table). Boat noise averaged 31% of the time in tidal regions, but only 2% in non-tidal areas. Similarly, boat sounds (all types combined) averaged 0.02 sounds/min and 0.21 sounds/min in non-tidal and tidal regions, respectively (P < 0.0001). Average soundscape percent time of traffic sounds and all anthropophonic sounds combined were also significantly different between river regions (traffic = 8.3% and 0.8%, noise = 12.1% and 33.2%, for non-tidal and tidal regions, respectively, both P < 0.05). Surface sounds were significantly more frequent, numerous, and occupied more time in tidal regions than non-tidal. No other biophonic sounds were significantly different among regions (S10 Fig, S6 Table).

### Ambient noise level

Overall received ambient SPL values ranged from 90 to 133 dB re 1 μPA rms. There was no significant difference in received ambient SPL among habitat types during the day with averages (± SE) of 99.4 (2.2), 98.7 (1.2) and 101.1 (1.4) dB re 1 μPA rms, for brook/creek, pond/lake and river habitats, respectively (based on a one-way ANOVA on log transformed data; N = 77). However, average power spectral density curves of the ambient noise suggest differences in the frequency structure among habitat types (S6 Fig). Brook/creek habitats tend to have the highest levels and pond/lake habitats the lowest levels at frequencies below 500 Hz, while river habitats have the highest levels at all higher frequency bands.

**Fig 5:**
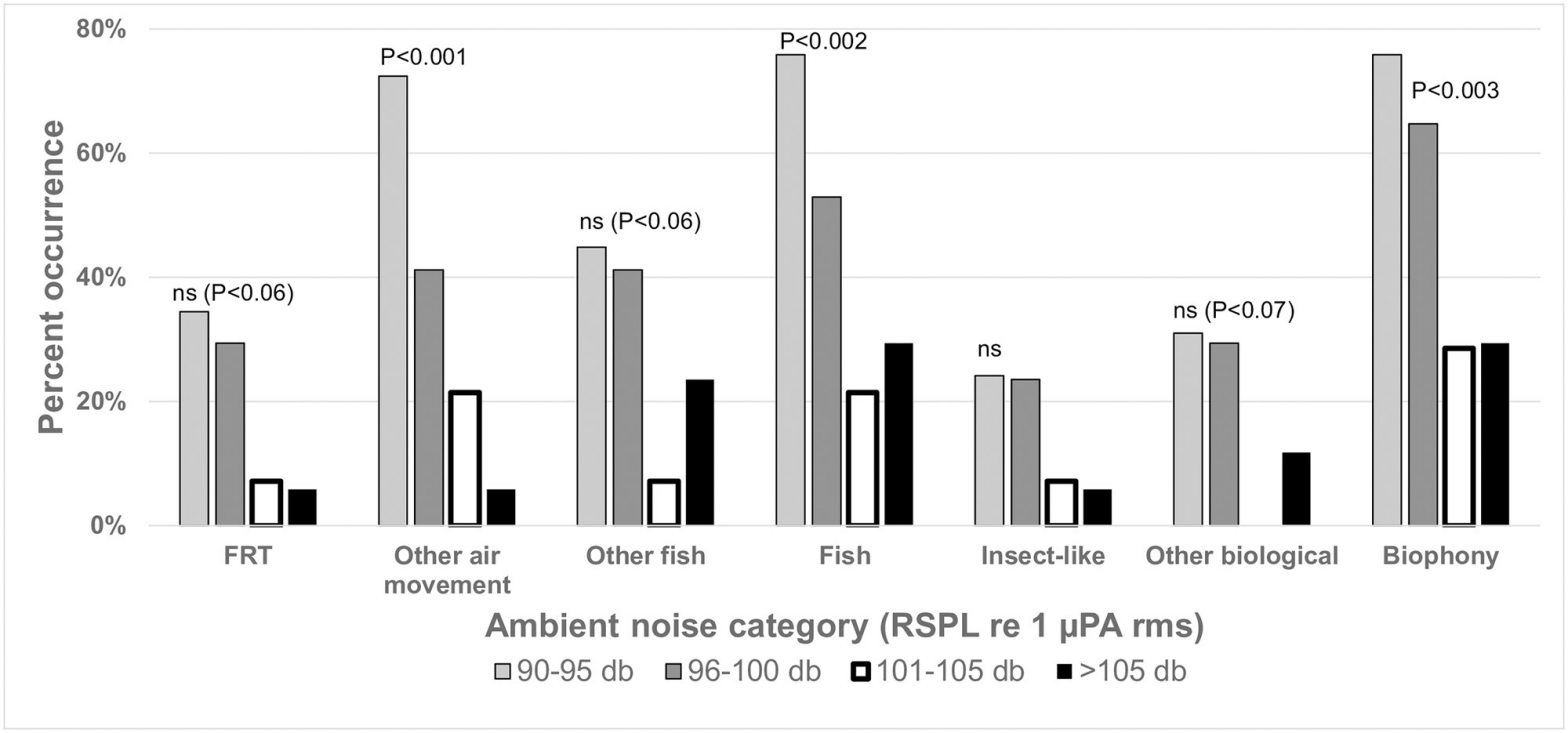
Ambient noise level effect. Comparison of the frequency of occurrence of selected biophony sound categories among locations grouped into four ambient noise categories: 90–95 dB (N = 29), 96–100 dB (N = 17) 101–105 dB (N = 14) and >105 dB (N = 17). A Chi Square (Chisq) test in a one-way contingency table of frequencies tested for differences in the observed group frequency from the expected group frequency (P ≤ 0.05, ns = not significant). See S7 Table for details.

There were significant differences in the biophony among the four ambient noise categories (Fig 5, S7 and S8 Tables). Air movement sounds significantly declined from a high of 72% of locations to a low of 6% of locations from the lowest to the highest ambient noise level categories. Similar, but non-significant, trends in occurrence were observed for FRT and other fish sounds (Fig 5). Rate and percent time of biological sounds followed similar trends with significant declines in air movement, fish and total biophony with increasing ambient SPL (S8 Table). There was a highly significant decline in the standardized diversity of biophonic sound types from 3.2 (± 0.6) to 0.1 (± 0.1) sounds from the lowest ambient noise to the highest ambient noise category (S12 Fig).

**Fig 6:**
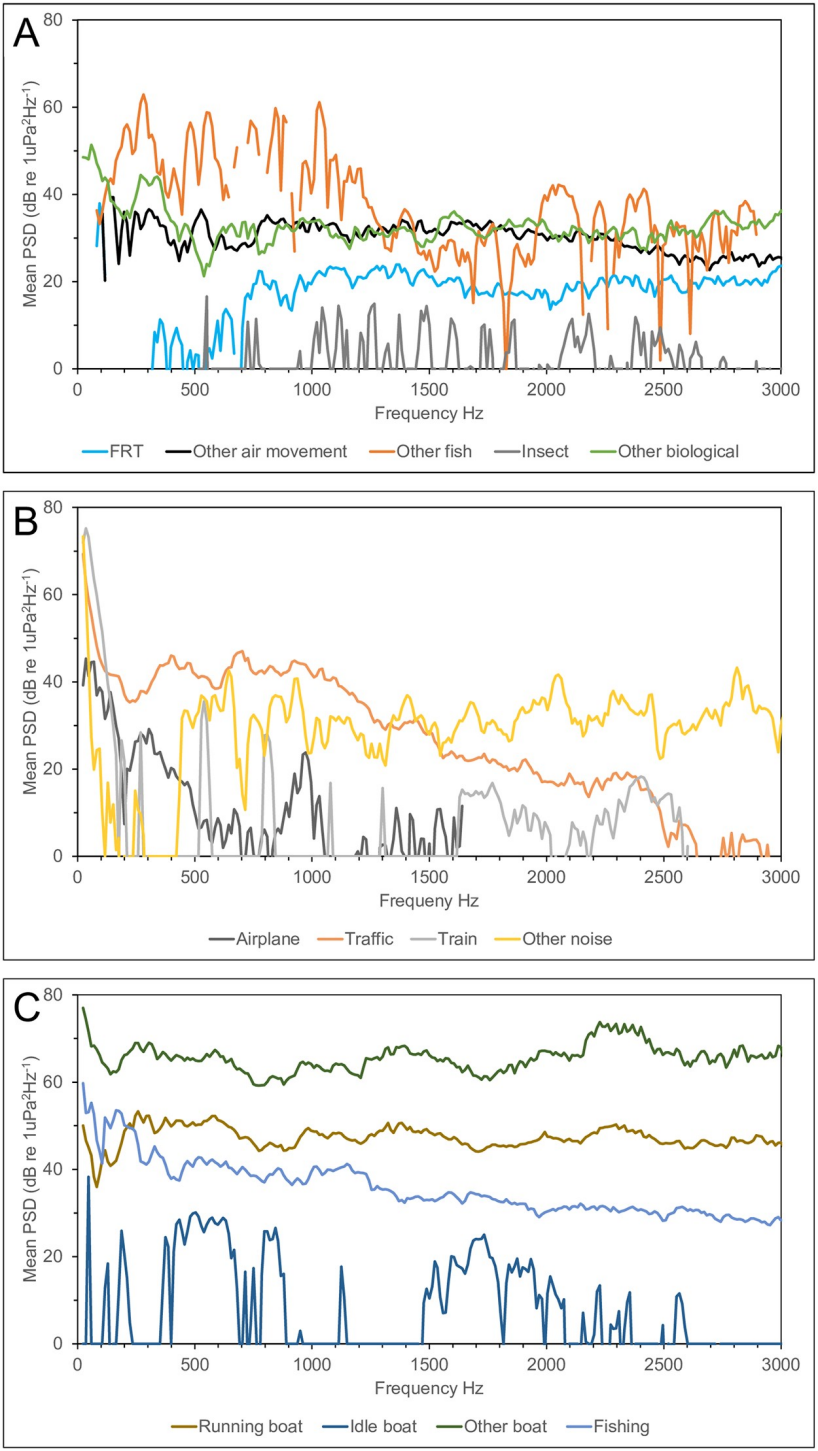
Selected biophony and anthrophony spectra above local ambient noise. Mean power spectral density (PSD) curves above the ambient for the biophony (A) and anthropophony (B and C) were calculated as the received level difference between individual sounds and the ambient spectra from the same recording and then averaging among all subsampled sounds within each biophonic category (Hanning, FFT 4096, 50% overlap, frequency resolution 11.7, PSD normalized).

A comparison of the average power spectral density curves of major biophony categories with major anthropophony categories indicates that other fish sounds are above average anthropogenic sounds, except for running and other boat sounds (S6 Fig). There is also considerable overlap with the spectra of traffic sounds. Air movement and insect sounds produce low amplitude sounds largely below anthropogenic sound levels and ambient spectra averaged across all recordings (S6 Fig). Air movement sounds nevertheless exhibit significant energy above ambient noise from the locations they occurred at, while insect-like sounds tended to be close to ambient levels (Fig 6).

## Discussion

The three most significant findings in this study are: 1) freshwater habitats in the New England region of North America contain a diverse array of unidentified biological sounds; 2) air movement sounds constitute an important component of freshwater soundscapes; and 3) anthropogenic noises dominate the soundscape and their overlap in time and frequency with the biophony, together with their high sound levels, suggests a high potential for negative impacts. The impact of anthropogenic noise on the natural freshwater soundscape is most strongly illustrated in Fig 4 which demonstrates the prevalence of the anthropophony accounting for 90% of the soundscape by relative mean percent time. This finding alone signals how dramatically freshwater soundscapes have been modified by human-produced noise. The strong spectral overlap between the biophony and anthropophony further hints at potential impacts (Fig 2). However, masking only occurs when individual biophonic sounds overlap in time and frequency with individual anthropophonic sounds. Assuming no bias in detection, high rates of temporal overlap with the biophony were only found with traffic noise (ranging for 4% to 11% depending on the biophonic category), while if detection was biased, then the overlap is underestimated. It should be pointed out that any sound level of anthropophonic noise in these habitats alters the soundscape from that in which aquatic organisms have evolved. The potential for impact is further supported by the observed higher sound levels of anthropophony sources compared to biophony sources (Fig 6 and S6 Fig). Finally, the highly significant decline in the frequency of occurrence (Fig 5, S7 Table), number and percent time of sounds (S8 Table), and diversity (S12 Fig) of the biophony with increasing ambient received sound level suggests that high noise levels, whether natural or anthropogenic, can affect sound production and soundscape composition (although bias from masking of sound detection must be considered as discussed below).

Our observations of the widespread occurrence of air movement sounds in many habitats across a large geographic area, together with their large contribution to the biological soundscape based on sound rate and sound percent time, suggest for the first time that air movement sounds are an important phenomenon in multiple types of freshwater habitats. We emphasize that even if air movement sounds are largely incidental, if sounds are species specific [45], they can be used by scientists and resource managers as an aid in documenting the spatial and temporal distribution of fishes and their associated soniferous behavior [49]. In addition, we point out that even incidental sounds can expose an organism to predation by organisms that can hear that sound, and hence would be subject to natural selection pressures.

Despite the lack of significant habitat differences in ambient noise levels, sound level appeared to have a strong negative influence on biological diversity and soundscape composition (Fig 5, S7 and S8 Tables), suggesting possible masking, suppression of sound production, or avoidance of locations with high ambient noise levels regardless of their source. A negative effect of ambient noise level on biological sound production or detection supports previous work on potential impacts of noise levels on freshwater fishes [e.g. 10–11, 13, 20, 25, 37 and review in 7], however we caution that the results do not necessarily suggest the observed trend was due to purely anthropogenic noise effects since no biological sounds were negatively correlated with any noise rate or percent time variable. The lack of such correlations may simply be due to the high diversity of sounds observed while sampling over a wide variety of habitats and geographies with many different faunal assemblages. The overlapping frequency structure of anthropogenic noises with the biophony, especially with the other fish category (Fig 2, S6 Fig, Table 1) also suggests the potential for masking (an example of which can be seen in S2 Fig and the corresponding S2 Audio). In contrast, the lack of overlap between peak frequencies of biological sounds and peak ambient frequency (Fig 2, S6 Fig) lends further support to previous studies suggesting that fish take advantage of acoustic niches [e.g. 25]. Our data suggest that a high-frequency “quiet noise window” may also occur in other freshwater habitats, and that freshwater organisms may have evolved to exploit different acoustic windows in different habitats. It is also possible that multiple acoustic windows may occur in some habitats which can be exploited by different organisms. The impact of anthropogenic noise on the natural noise windows in various habitats remains poorly understood.

Running boats generate long-lasting noise starting with low-amplitude and low-frequency noise while in the distance and progressing to high-amplitude noise that saturates the recordings when within a few hundred meters (depending on the boat type and speed), before gradually fading as the boat moves off in the distance again (an example of the sound of an approaching boat which anchors within 100 m of the recording site is provided in S4 Fig and corresponding S4 Audio). Boat noise in excess of 90% time were observed at some locations (S1 Data set), highlighting the potential of boat noise as a masking source in freshwater. Similar observations have been reported in freshwater [32] and marine habitats [e.g. 50–52]. Smott et al [51] reported three types of boat sounds: burst broadband, variable broadband, and low frequency, which likely correspond with our other boat, running boat, and idle boat categories. Locations near important navigation routes, marinas, or boat landings experience chronic boat noise exposure. In some cases, boat noise is so prevalent in the hours around sunset (when boaters are returning to shore) that recording of biological sound is nearly impossible. Our sampling was conducted during the early season when boating activity was sparse and many boat ramps had not yet opened for the season. We would expect, therefore, that boat noise would be significantly more prevalent during the summer months than that observed in our study, similar to reports from marine systems [e.g. 50–52]. Boat noise was undoubtedly an important chronic noise source in navigable waters where it dominated the soundscape and unquestionably masks many biological sounds (Fig 6, S4 and S6 Figs). Boat noise is particularly problematic in enclosed water bodies such as small lakes, and in linear rivers as the sound travels great distances. We have often detected motor boat sounds before sighting the vessel in the distance. On the other hand, serpentine waterways may be less impacted because sounds of an approaching vessel are blocked by land until the vessel moves around the bend and into the line of sight.

Our observations suggest that boats running idle while docked, anchored, or drifting are a major component of freshwater soundscapes (Fig 4), and have the potential to mask some biophony such as insect sounds, but we are not aware of studies that examine its potential impact in freshwater. However, at least one study in a small estuarine river reported boat sounds attributed to idling to be important masking sources for sciaenid fishes [51]. The tendency for boaters, and especially ferries and other large vessels, to idle for long periods, and the lower frequency structure of idling boats (Figs 2 and 6, S6 Fig) suggests that boat idling may be an important chronic noise source in navigable waters. Power density spectra of boat sounds in this study were remarkably similar to those reported in other freshwater [e.g. 10, 42] and marine studies [e.g. 51].

Traffic noise exhibits similar though much less extreme wax-and-wane patterns as can clearly be seen in S2 Fig and heard in the corresponding S2 Audio. Our observations support those of previous researchers that traffic sounds can be important in some freshwater habitats [34–38, 41–42]. Holt and Johnston [37] measured traffic noise propagation distances in streams and concluded that traffic sounds may have negative impacts in freshwater habitats. In contrast, ice road traffic (with similar signatures to our traffic sounds) were not thought to impact burbot (*Lota lota*, Gadidae) under the ice in Great Slave Lake, Canada [41]. Hopson [38] found evidence of traffic impacts on both the above and below water biophony in freshwater wetlands of Ohio.

Although not as extreme as boat noise, traffic sounds are far more ubiquitous in freshwater habitats and can be chronic in urban areas and during rush hours. In fact, the increase in traffic contribution to the soundscape at night was due in part to our sampling around sunset when traffic tends to be heavy. The relatively high temporal overlap between biological sounds and traffic sounds suggests a high likelihood for impacts, especially for other fish sounds which had both high temporal (10%) and acoustic frequency overlap (Figs 2 and 6, S6 Fig). A comparison of the power density spectra of traffic sounds with the biophony (Fig 6, S6 Fig) suggests they would likely mask all but the other fish category. Although we did not examine propagation distances of traffic sounds, traffic crossing bridges was detected in locations as far as 270 m from the nearest bridge (S1 Data set), agreeing well with previous studies [37, 42]. Differences in the relative contribution of traffic and boat noises to the soundscape among habitats and river regions demonstrate variations in potential noise impacts among habitats and river zones (S9 and S10 Figs, S5 and S6 Tables). It should be recognized that traffic sounds were likely over-represented in our sampling due to the frequent necessity of accessing the water near bridges. However, as has previously been pointed out [37], in many areas bridge crossings are common and smaller streams and rivers may be crossed many times over short stretches in urban and suburban locations.

The inconsistent trends in observations moving along the river main stem locations likely result from the fact that river systems form a coenocline where gradients in abiotic and biotic conditions regulate community structure and habitat function [53]. Thus, comparison among river systems of different lengths and elevation gradients requires a gradient approach. To our knowledge this is the first study to examine river order changes in freshwater soundscapes, although differences in ambient noise levels have been compared among rivers [17, 19] and along short reaches of rivers [18]. Major differences in the soundscape were previously found between two locations in the Hudson River [31]. The only other study to examine the biophony of multiple locations within the same freshwater river, found significant differences among tributaries classified by degree of connectivity [22].

It is interesting that while the biotic community changes considerably from high elevation reaches to estuarine reaches, the changes in the biophony type contribution to the soundscape are minimal, suggesting that although soniferous species may change, the broad sound categories are more consistent. Classification of sounds to more specific sound types would likely have resulted in significant differences in the soundscape along the river coenocline due to species assemblage changes as it has been shown in a series of studies in a small estuarine river [51, 54–56]. A gradient in impacts from different types of anthropogenic noise is expected as we observed a striking transition from remote wilderness to increasingly developed urban areas while traveling from the river headwaters to the sea. Some of this transition was captured in the comparison between non-tidal and tidal reaches of the rivers where there was a shift between dominance of the soundscape by traffic noise in non-tidal reaches to a dominance by boat noise in tidal reaches (S10 Fig, S6 Table), highlighting different potential for impacts in different habitats.

Studies on the biophony of temperate freshwater habitats have used a variety of methodologies from point sampling to long-term sampling (S1 Table). Studies in rivers have reported 21 [31], 25 [26], and 128 [22] biophonic sound types. Similar numbers have been reported for ponds (48 [4]) and lakes (26 [43]). Except in single species studies, in most cases biophonic sound sources are unknown. As we pointed out in our review of sound production in temperate freshwater fishes [9], there is a critical need for studies to describe fish sound sources in freshwater habitats. Our use of real-time monitoring resulted in the detection of a high diversity of air movement sounds that would likely have been overlooked if the recordings had only been analyzed in post-processing (see S1 Audio for an example). In addition, real-time monitoring allowed us to identify false biophonic sounds such as creaking logs, twig rattling, and floating dock squeaking that appear very similar to biophonic sounds in their acoustic characteristics.

Freshwater habitats are highly impacted by terrestrial and aerial sound sources. Bird sounds are one component of the soundscape that may have important effects on fish behavior. For example, herring gull sounds are clearly audible underwater in herring runs, and are likely audible to alewife [45]. Other terrestrial sounds have similar potential ecological interactions. We advocate for studies that simultaneously record above and below water sounds [38–40, 57] to distinguish between above and below water sound sources, but ultimately to describe what we refer to as the “holo-soundscape” of freshwater systems. Kuehne et al [40] attempted to correlate above and below water sound recordings, but unfortunately defined the biophony as sounds from 3–8 kHz and the anthropophony as sounds of 1–2 kHz which confounds the two sound sources, given that many fish sounds would fall in the lower frequency bandwidth. Hopson [38] reported higher sound intensity, more anthropophonic sound occurrences, and lower acoustic diversity in both above and below water wetland soundscapes in disturbed versus non-disturbed areas.

Recent developments in acoustic sensor technology (e.g. long performance dataloggers) and of automated processing methods (e.g. automatic recognition methods and acoustic indices) now allow researchers to relatively inexpensively collect long-term recordings and to process them in timeframes which are unattainable by more traditional recording and analysis approaches (e.g. manual and aural quantification of sound occurrences) [4]. These developments are rapidly expanding the field of passive acoustic research and have the potential to quickly identify biophonic patterns and anthropophonic impacts on them [4]. However, as the biophonic component of most aquatic habitats is still poorly understood, the application of automated processing methods alone, without a prior knowledge of the type of signals present in a specific site, might result in interpretations that do not accurately reflect the biodiversity and the ecological status of the area. Efforts to identify specific sound sources in freshwater soundscapes are, therefore, critically needed. Attempts to correlate unknown sounds with the presence of macroinvertebrates (e.g. [13, 22, 35–36]) or fishes [2, 23–24, 27, 32] are especially helpful. Follow-up studies that attempt to identify the unknown sound sources are also important (e.g. [23, 31, 45]). Unfortunately, few studies incorporate real-time monitoring of sounds (S1 Table, [2, 23, 27, 31]). A series of studies in the estuarine May River of South Carolina [51, 54–56] demonstrate the advantage of having well documented sound sources when attempting to examine anthropophonic impacts and ecological influences on the underwater soundscape. Whenever possible when beginning a research program in previously unstudied freshwater habitats, researchers should attempt to conduct preliminary studies utilizing real-time sound monitoring and visual observations of the holo-soundscape, together with faunal sampling and field-auditioning of aquatic organisms before, or concurrently with, the collection of long soundscape sound series.

## Supporting information

S1 Fig Representative sampling sites. A) Brook wp 42, B) Creek wp 60, C) Pond wp 68, D) Lake wp 83, E) Tributary wp 117, F) River wp 101. The waypoint (wp) number can be used to look up the location details in the S1 Data set. The individual pictured in S1Fig E has provided written informed consent (as outlined in PLOS consent form) to publish their image alongside the manuscript.
(TIF)

S2 Fig Example of traffic sound. Relative amplitude (top) and spectrogram (bottom) of traffic and fish sounds recorded at night on 14 May 2008 in Sucker Creek, Griffin, New Hampshire (N43° 00.257’ W71° 20.933’). As a car passes over a nearby bridge (Traffic), catfish sounds (Other Fish) are partially masked. An unmasked catfish sound occurs later in the clip and indicates a true peak frequency well within the traffic noise. Examples of a fish splashing at the surface (Surface), and subsequent air movement (Other Air movement) and FRT sounds can also be seen relative to the traffic sound. An amplified audio file corresponding to the figure can be heard in S2 Audio online. Spectrogram parameters: unfiltered, 1,024-point Hann windowed FFTs with 50% overlap.
(TIF)

S3 Fig Example of train noise. Recorded on 3 May 2008 in Deep River, a tributary of the Connecticut River in Deep River, Connecticut (N41° 22.978’ W72° 25.566’). Top: photograph of the train passing by at the time of the recording. Bottom: relative amplitude waveform and spectrogram of the train sound which can be heard in the corresponding S3 Audio online. Spectrogram parameters: unfiltered, 1,024-point Hann windowed FFTs with 50% overlap.
(TIF)

S4 Fig Example of running boat noise. The sound of an outboard motor boat as it approaches from the distance and stops to anchor nearby, recorded on 3 May 2008 in the mainstem of the Connecticut River in Old Saybrook, Connecticut (N41° 19.143’ W72° 21.028’). Top: Photograph of the boat as it passes, Bottom: relative amplitude waveform and spectrogram of the noise generated by the passing boat, which can be heard in the corresponding S4 Audio online. Spectrogram parameters: unfiltered, 1,024-point Hann windowed FFTs with 50% overlap.
(TIF)

S5 Fig Example of other boat noise. Sound produced by the power trim of a nearby outboard boat, recorded on 1 May 2008 in the mainstem of the Connecticut River in Northampton, Massachusetts (N42° 20.114’ W72° 37.211’). The corresponding sound can be heard in the S5 Audio online. Spectrogram parameters: unfiltered, 1,024-point Hann windowed FFTs with 50% overlap.
(TIF)

S6 Fig Average power spectral density of major sound categories. Power spectral density (PSD) averaged over a subsample of sounds from each major sound category (samples sizes are shown in S3 Table). A) selected biophonic sounds, B and C) selected anthropophonic sounds, D) ambient noise from each habitat category. Spectrogram parameters: Hanning, FFT 4096, 50% overlap, frequency resolution 11.7, PSD normalized.
(TIF)

S7 Fig Comparison between day and night soundscapes. Major components of the soundscape: A and C) during the day and night, respectively, based on mean number, B and D) during day and night, respectively, based on mean percent time. The relative contribution of the biophony compared to the anthropophony is shown in the main pie, while the composition of the biophony slice is shown in the expanded pie. The size of the wedge represents the relative proportion of the sound out of all sounds based on data found in Table 2 of mean number per minute (A and C) and mean percent time (B and D).
(TIF)

S8 Fig Hourly trend in biophony abundance. Mean number of sounds of major biophony categories by hour of the day.
(TIF)

S9 Fig Comparison of habitat soundscapes. Comparison of soundscapes among habitat categories and diel period based on mean percent time. Day: A) creek/brook habitat during the day (N = 21), B) pond/lake habitat (N = 41), C) river habitat (N = 79). Night: D) creek/brook habitat (N = 2), E) pond/lake habitat (N = 7), F) river habitat (N = 15). See S5 Table for summary statistics.
(TIF)

S10 Fig Comparison of soundscapes between tidal and non-tidal river regions. Relative contribution of anthropophony and biophony to the aquatic soundscape of non-tidal (top) and tidal (bottom) main-stem river regions during the day based on mean number of sounds per minute (A and B) mean percent time (C and D) of each sound type. Data and statistics are provided in S6 Table, while the size of the wedge represents the relative proportion of the sound out of all sounds.
(TIF)

S11 Fig Average day-time soundscape composition. Venn diagram illustrating the relative composition of the anthropophony and biophony and their major constituents to the day-time soundscape. The diameter of each circle is proportional to the mean percent of recording time for the indicated sound category. Circles within large circles represent subcomponents of the larger category. For example, the fish category contains two nearly equal subcomponents (other fish and air movement sound).
(TIF)

S12 Fig Decline of biophony diversity with increasing ambient noise level effect. Comparison of the number of biophony sound types among locations grouped into four ambient noise levels based on received sound pressure level (RSPL). The decline in diversity form low to high noise level is highly significant (P ≤ 0.001) during the day (gray bars, N = 74), but nonsignificant during the night (black bars, N = 16).
(TIF)

S1 Table Selected review of passive acoustic studies in temperate freshwater habitats with a few comparative marine studies. Brief description of soundscape studies in temperate freshwater systems. Type = is the general type of study. Effort = brief description of sampling regime. Lake, Pond, River, Stream, Other = the number of unique habitats sampled (i.e., specific river or lake). The total number of specific sites within the habitat type is given in parenthesis. N = total sample size when known. Anthropophony = indicates some attention was given to describing anthropogenic sound sources. Biophony = when some quantification or description of the biophonic composition is provided. Real-time = sampling included some real-time listening/observations of soundscape. Other data = other types of data collected to correlate to soundscape data.
(XLSX)

S2 Table Sampling effort for all locations. (XLSX)

S3 Table Subsample sizes used for spectral analysis by sound type. (XLSX)

S4 Table Percent occurrence of sounds at locations by habitat category. (P = significance level for a Chi Square (Chisq) test in a one-way contingency table on differences among habitats within a diel period, * = < 0.05, ** = < 0.01, *** = <0.001, n/a = no test; N = 148 for day and 25 for night).
(XLSX)

S5 Table Mean percent time by habitat category. Comparison of the mean percent time (total duration of sounds in the category divided by recording duration) of sound types among habitat types. SE = standard error of the mean. P = probability of a significant difference among habitats based on a one-way ANOVA on transformed variables, performed separately by diel period. (* = < 0.05, ** = < 0.01, *** = <0.001, n/a = no test).
(XLSX)

S6 Table Comparison of summary statistics between tidal and non-tidal river regions. Mean number per minute, percent of recording time per minute, and percent occurrence of sound categories for day sampling within tidal and non-tidal river regions (SE = standard error of the mean, P = significance level from a one-way analysis of variance, Chisq P = significance level from a Chi Square test of expected frequencies in a one-way contingency table, * ≤ 0.05, ** ≤ 0.01, *** ≤ 0.001).
(XLSX)

S7 Table Frequency of occurrence of biophony among ambient noise level categories. Comparison of the percent frequency of occurrence among day-time recordings grouped into received ambient noise level categories (RMS = root mean square). (P = significance level from a Chi Square test of difference among SPL categories from the expected frequency in a one-way contingency table. ns = not significant, * ≤ 0.5, ** ≤ 0.01, *** ≤ 0.001).
(XLSX)

S8 Table Comparison of the mean number and mean percent time among daytime ambient noise categories. (P = results of a one-way ANOVA on differences among SPL levels of transformed variables. ns = not significant, * ≤ 0.5, ** ≤ 0.01, *** ≤ 0.001).
(XLSX)

S1 Audio Example of fish jump and air movement sounds with voice notes. Recorded observation of a fish jump followed by air movement sounds, together with voice notes on the event. Recorded at dusk on 30 April 2008 in Deerfield River, West Deerfield, Massachusetts (N42° 31.591’ W72° 37.952’). The sound has been amplified for optimal listening online.
(MP3)

S2 Audio Traffic sound with biophony. Sound file corresponding to S2 Fig containing the sound of traffic noise and examples of other fish, other air movement and FRT sounds recorded at night on 14 May 2008 in Sucker Creek, Griffin, New Hampshire (N43° 00.257’ W71° 20.933’). The sound has been amplified for optimal listening online.
(MP3)

S3 Audio Train sound. Example of train noise corresponding to S3 Fig and recorded on 3 May 2008 in a tributary of the Connecticut River in Deep River, Connecticut (N41° 22.978’ W72° 25.566’). Note that several very faint other air movement sounds (chirps) can be heard in the recording but are high frequency and not shown in the S3 Fig. The sound has been amplified for optimal listening online.
(MP3)

S4 Audio Running boat sound. Example of the sound of an outboard motor boat, corresponding to S4 Fig, as it approaches from the distance and stops to anchor nearby. Recorded on 3 May 2008 in the mainstem of the Connecticut River in Old Saybrook, Connecticut (N41° 19.143’ W72° 21.028’). The sound has been amplified for optimal listening online.
(MP3)

S5 Audio Other boat noise. Example of noise produced by the power trim of a nearby outboard boat, corresponding to S5 Fig. Recorded on 1 May 2008 in the mainstem of the Connecticut River in Northampton, Massachusetts (N42° 20.114’ W72° 37.211’). The sound has been amplified for optimal listening online.
(MP3)

S1 Data set Meta-data of sounds observed in all recordings. Recording location meta-data with sound rates and percent time by sound category. The data set is in Excel format with two worksheets: 1) data, 2) field definitions.
(XLS)
