# Supplementary figures and images for: Temperate freshwater soundscapes: A cacophony of undescribed biological sounds now threatened by anthropogenic noise
Source: PLoS One. 2020 Mar 18;15(3):e0221842. doi: 10.1371/journal.pone.0221842 (PMC7080229; doi:10.1371/journal.pone.0221842)

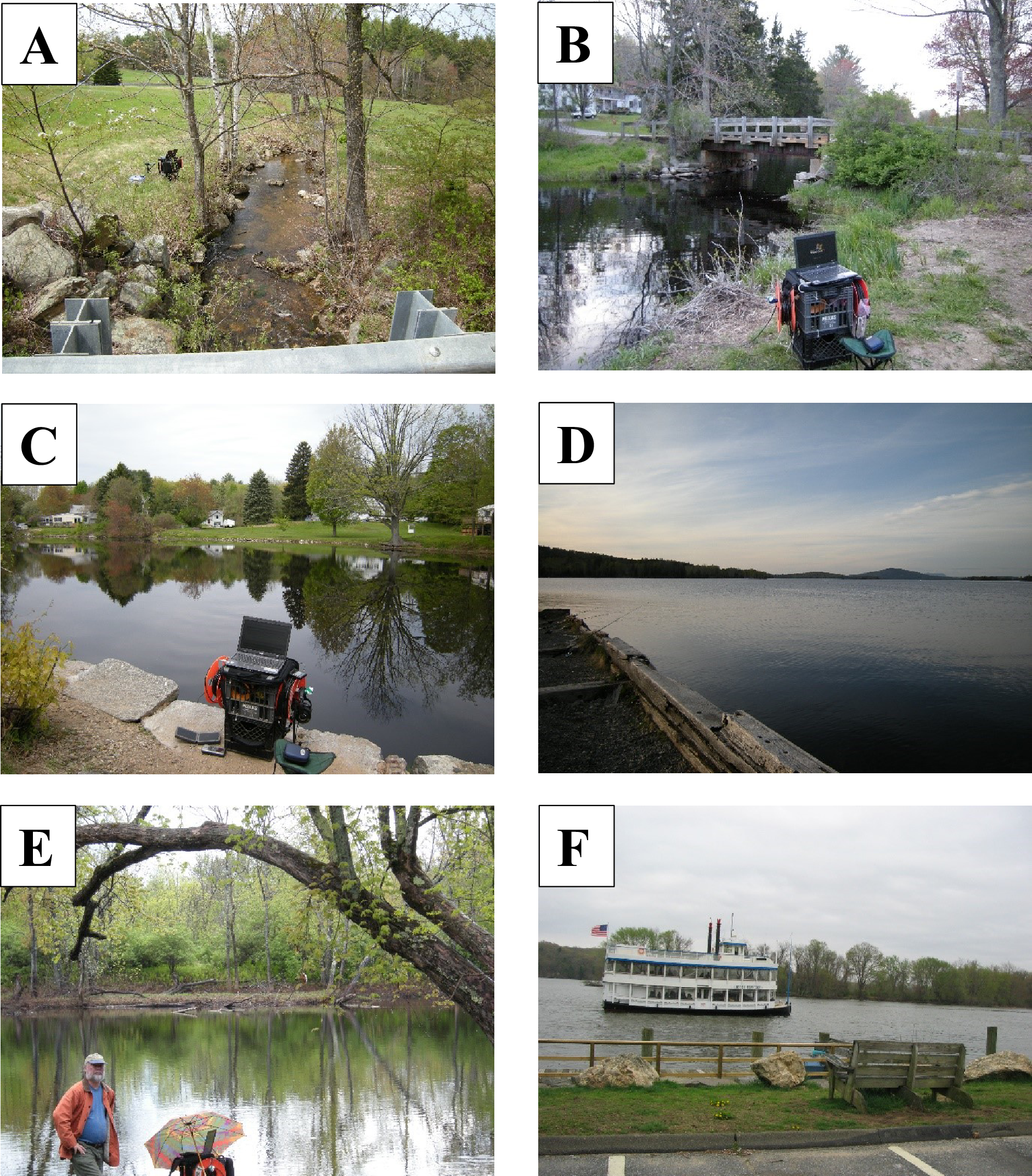

Supplement: S1 Fig — A) Brook wp 42, B) Creek wp 60, C) Pond wp 68, D) Lake wp 83, E) Tributary wp 117, F) River wp 101. The waypoint (wp) number can be used to look up the location details in the S1 Data set. The individual pictured in S1Fig E has provided written informed consent (as outlined in PLOS consent form) to publish their image alongside the manuscript. (TIF) [file pone.0221842.s001.tif]

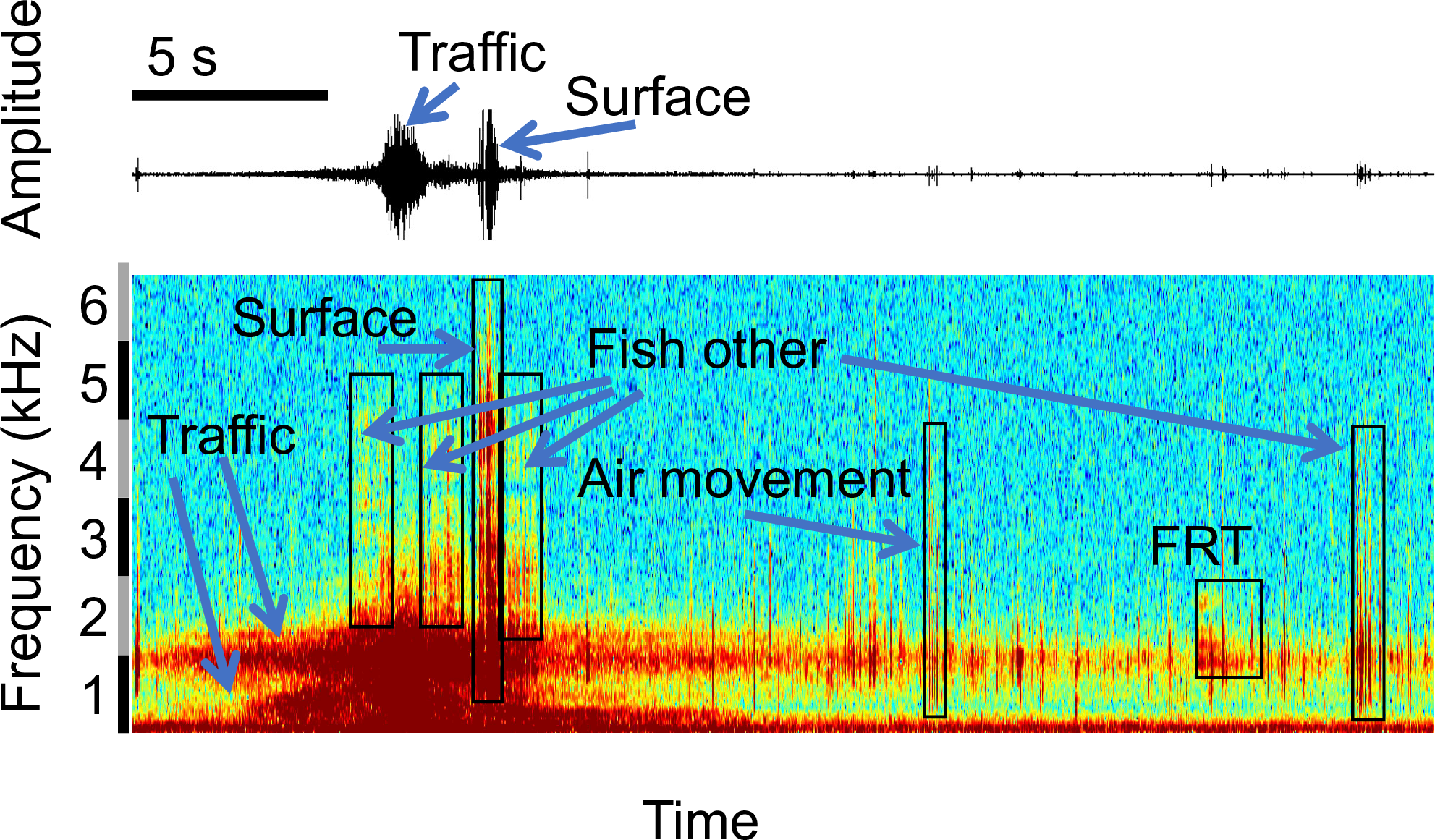

Supplement: S2 Fig — Relative amplitude (top) and spectrogram (bottom) of traffic and fish sounds recorded at night on 14 May 2008 in Sucker Creek, Griffin, New Hampshire (N43° 00.257’ W71° 20.933’). As a car passes over a nearby bridge (Traffic), catfish sounds (Other Fish) are partially masked. An unmasked catfish sound occurs later in the clip and indicates a true peak frequency well within the traffic noise. Examples of a fish splashing at the surface (Surface), and subsequent air movement (Other Air movement) and FRT sounds can also be seen relative to the traffic sound. An amplified audio file corresponding to the figure can be heard in S2 Audio online. Spectrogram parameters: unfiltered, 1,024-point Hann windowed FFTs with 50% overlap. (TIF) [file pone.0221842.s002.tif]

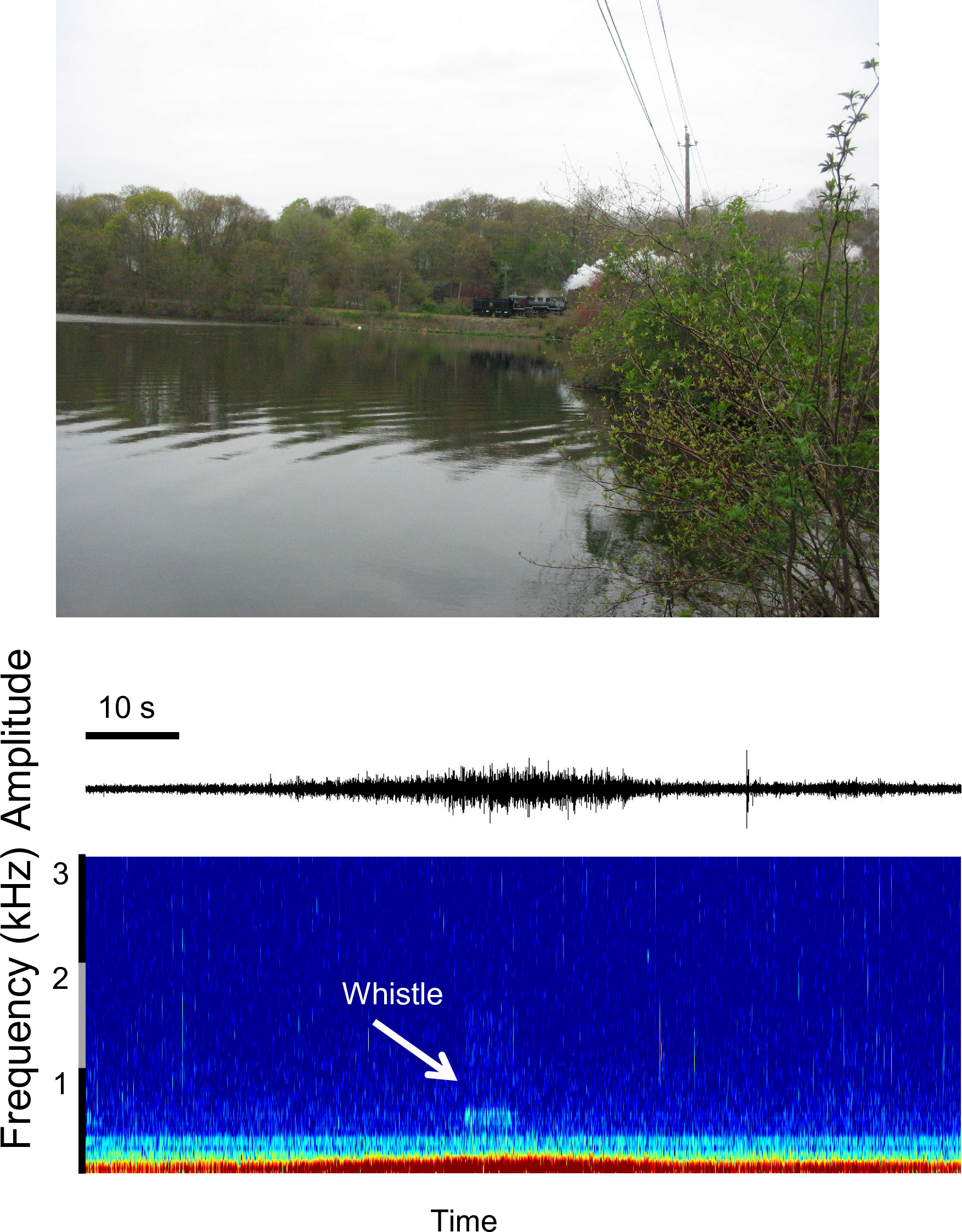

Supplement: S3 Fig — Recorded on 3 May 2008 in Deep River, a tributary of the Connecticut River in Deep River, Connecticut (N41° 22.978’ W72° 25.566’). Top: photograph of the train passing by at the time of the recording. Bottom: relative amplitude waveform and spectrogram of the train sound which can be heard in the corresponding S3 Audio online. Spectrogram parameters: unfiltered, 1,024-point Hann windowed FFTs with 50% overlap. (TIF) [file pone.0221842.s003.tif]

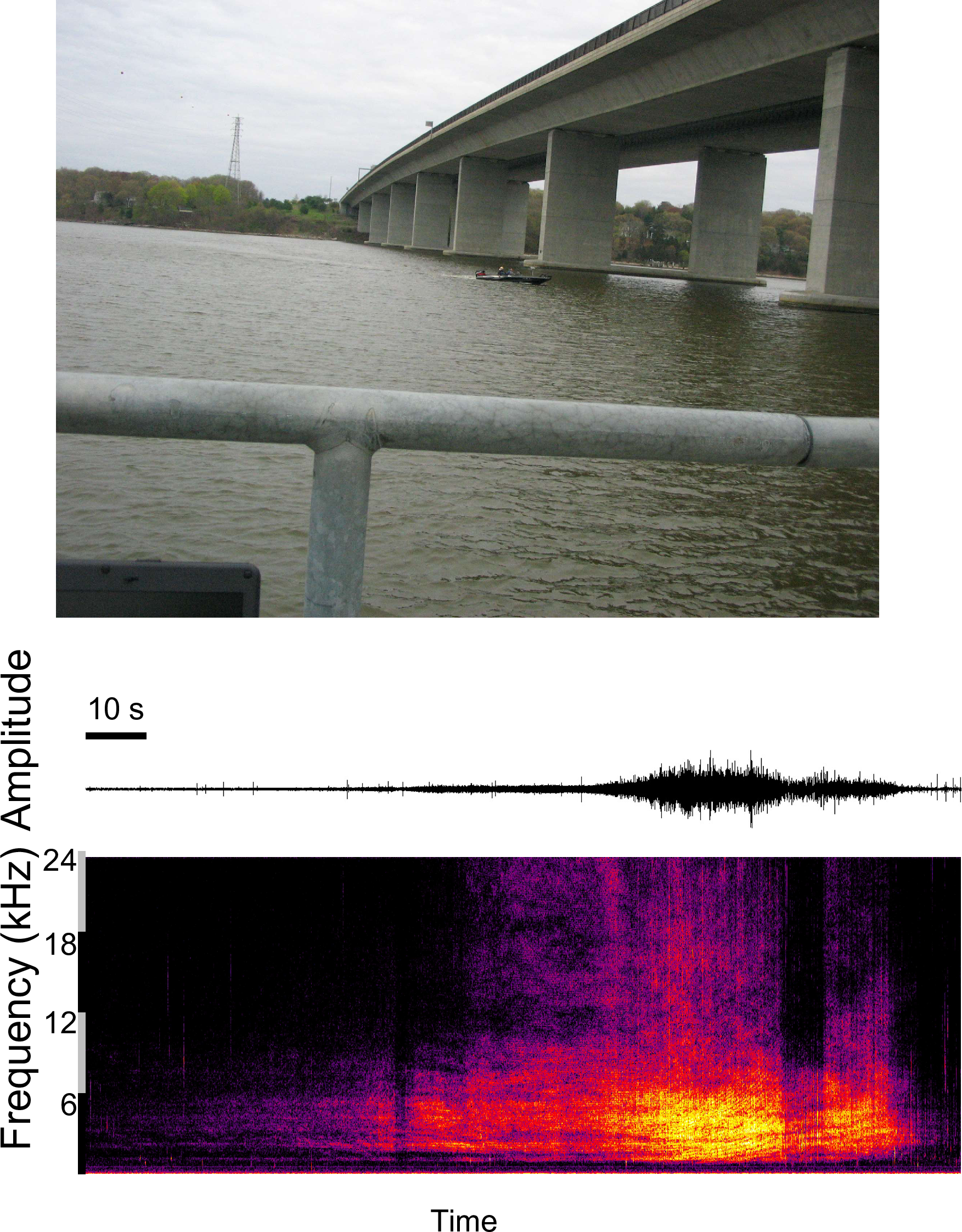

Supplement: S4 Fig — The sound of an outboard motor boat as it approaches from the distance and stops to anchor nearby, recorded on 3 May 2008 in the mainstem of the Connecticut River in Old Saybrook, Connecticut (N41° 19.143’ W72° 21.028’). Top: Photograph of the boat as it passes, Bottom: relative amplitude waveform and spectrogram of the noise generated by the passing boat, which can be heard in the corresponding S4 Audio online. Spectrogram parameters: unfiltered, 1,024-point Hann windowed FFTs with 50% overlap. (TIF) [file pone.0221842.s004.tif]

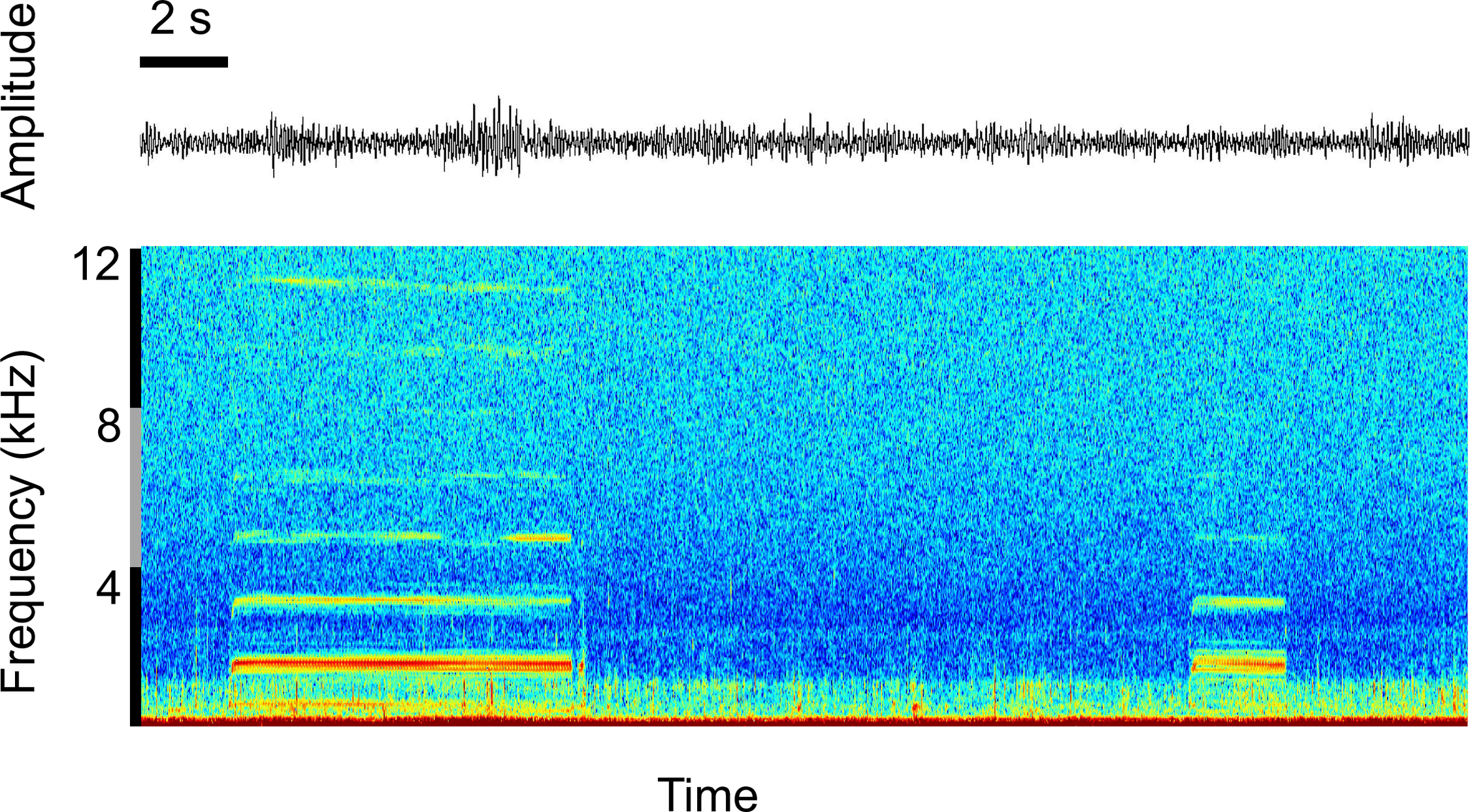

Supplement: S5 Fig — Sound produced by the power trim of a nearby outboard boat, recorded on 1 May 2008 in the mainstem of the Connecticut River in Northampton, Massachusetts (N42° 20.114’ W72° 37.211’). The corresponding sound can be heard in the S5 Audio online. Spectrogram parameters: unfiltered, 1,024-point Hann windowed FFTs with 50% overlap. (TIF) [file pone.0221842.s005.tif]

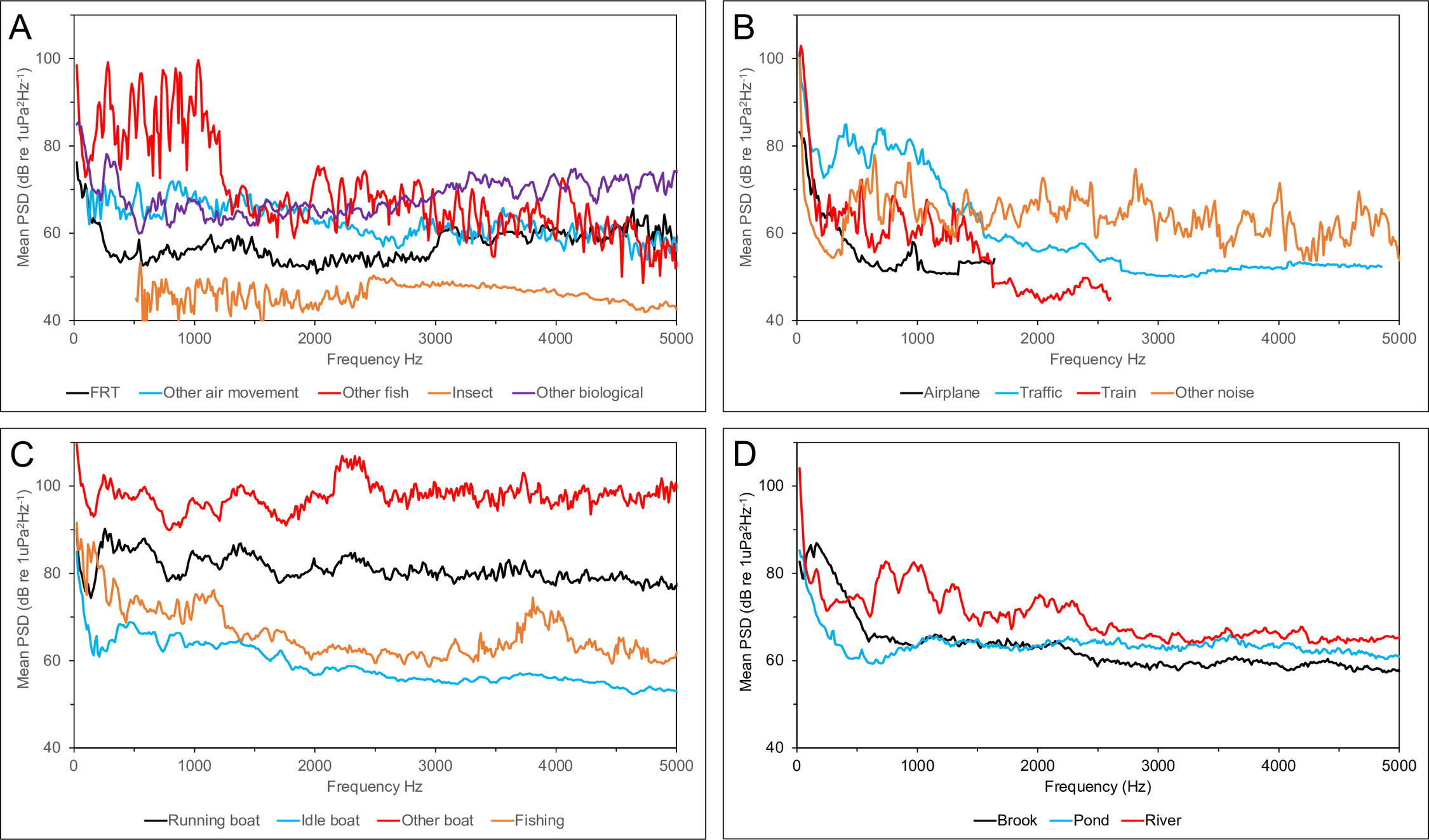

Supplement: S6 Fig — Power spectral density (PSD) averaged over a subsample of sounds from each major sound category (samples sizes are shown in S3 Table). A) selected biophonic sounds, B and C) selected anthropophonic sounds, D) ambient noise from each habitat category. Spectrogram parameters: Hanning, FFT 4096, 50% overlap, frequency resolution 11.7, PSD normalized. (TIF) [file pone.0221842.s006.tif]

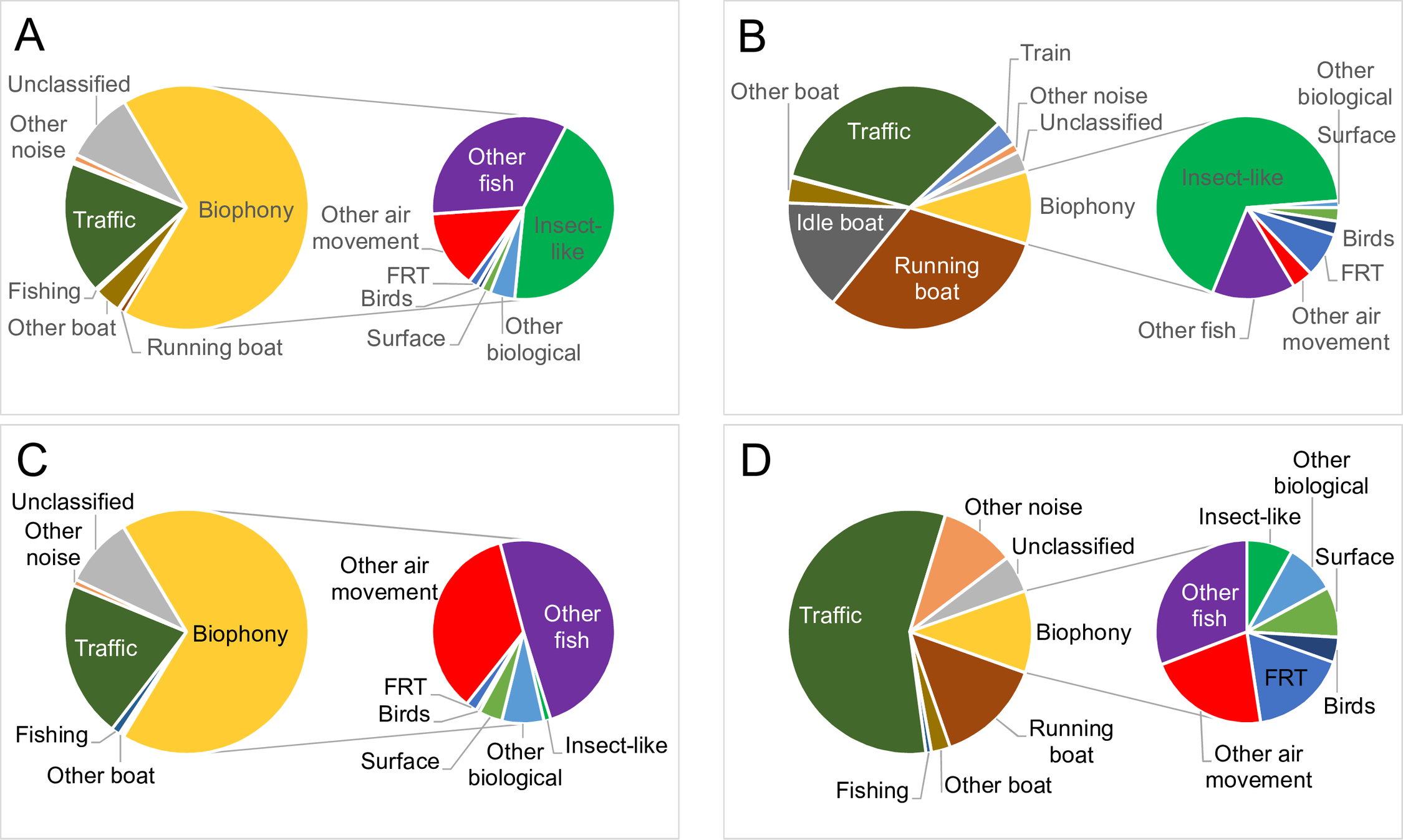

Supplement: S7 Fig — Major components of the soundscape: A and C) during the day and night, respectively, based on mean number, B and D) during day and night, respectively, based on mean percent time. The relative contribution of the biophony compared to the anthropophony is shown in the main pie, while the composition of the biophony slice is shown in the expanded pie. The size of the wedge represents the relative proportion of the sound out of all sounds based on data found in Table 2 of mean number per minute (A and C) and mean percent time (B and D). (TIF) [file pone.0221842.s007.tif]

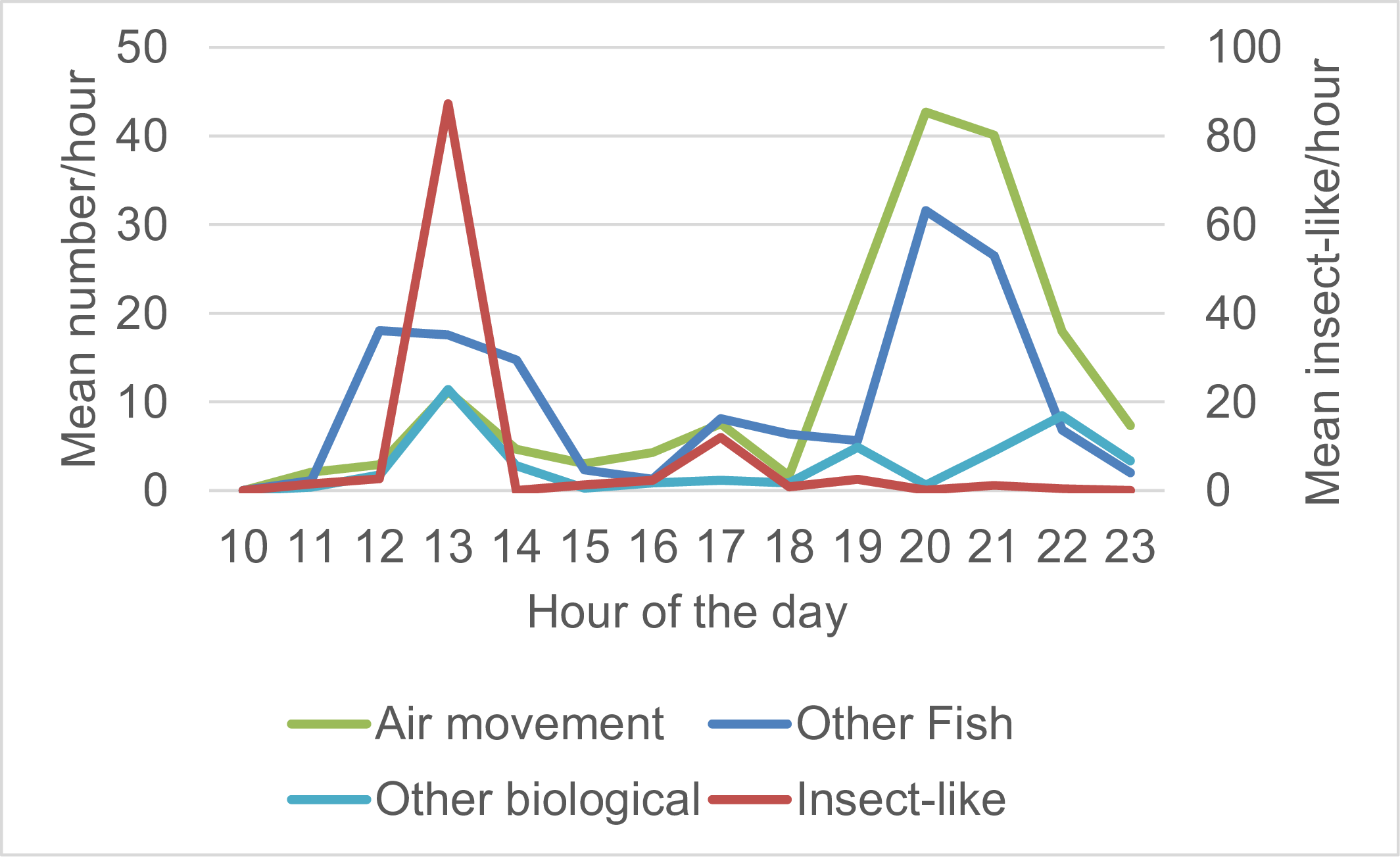

Supplement: S8 Fig — Mean number of sounds of major biophony categories by hour of the day. (TIF) [file pone.0221842.s008.tif]

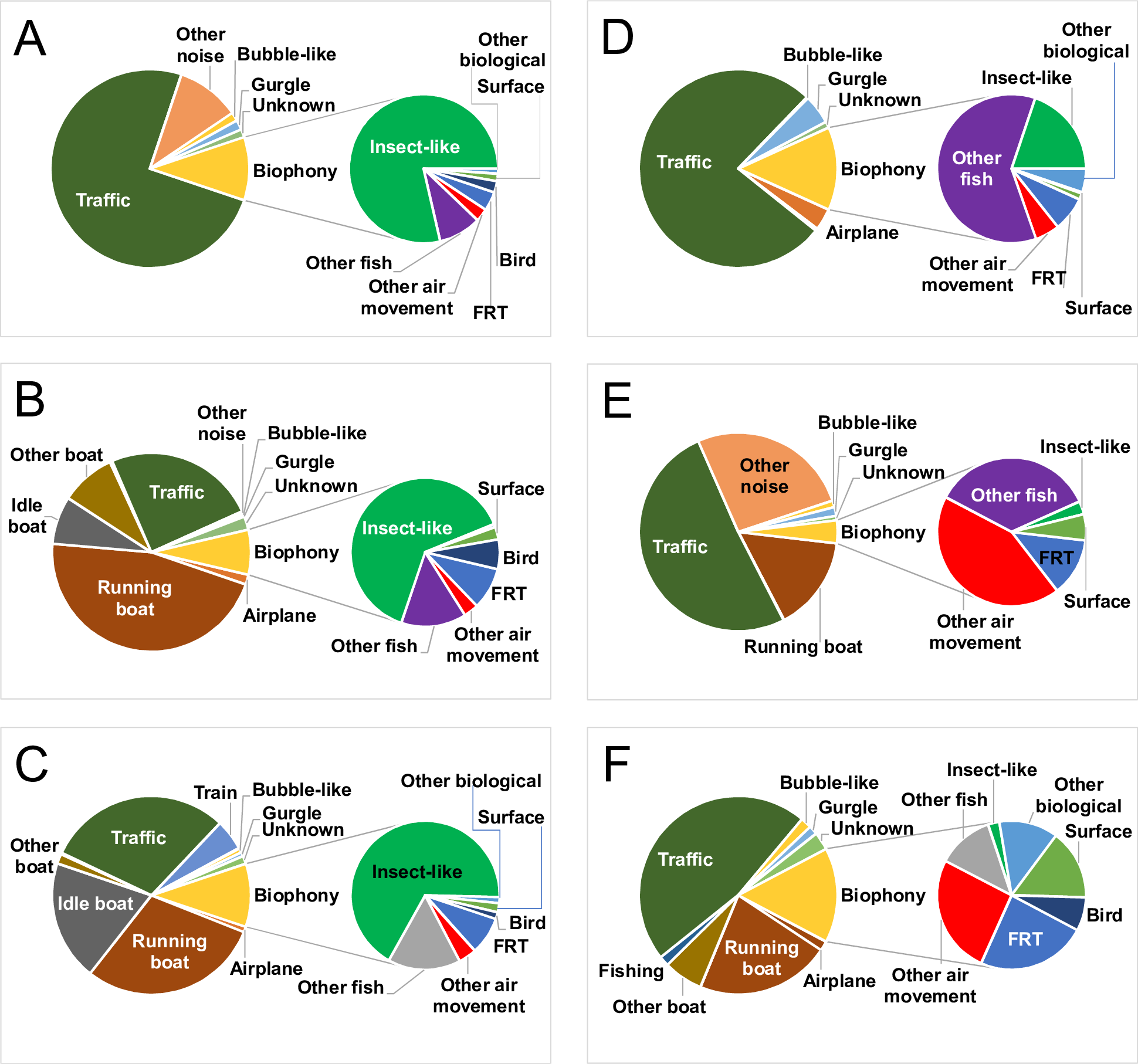

Supplement: S9 Fig — Comparison of soundscapes among habitat categories and diel period based on mean percent time. Day: A) creek/brook habitat during the day (N = 21), B) pond/lake habitat (N = 41), C) river habitat (N = 79). Night: D) creek/brook habitat (N = 2), E) pond/lake habitat (N = 7), F) river habitat (N = 15). See S5 Table for summary statistics. (TIF) [file pone.0221842.s009.tif]

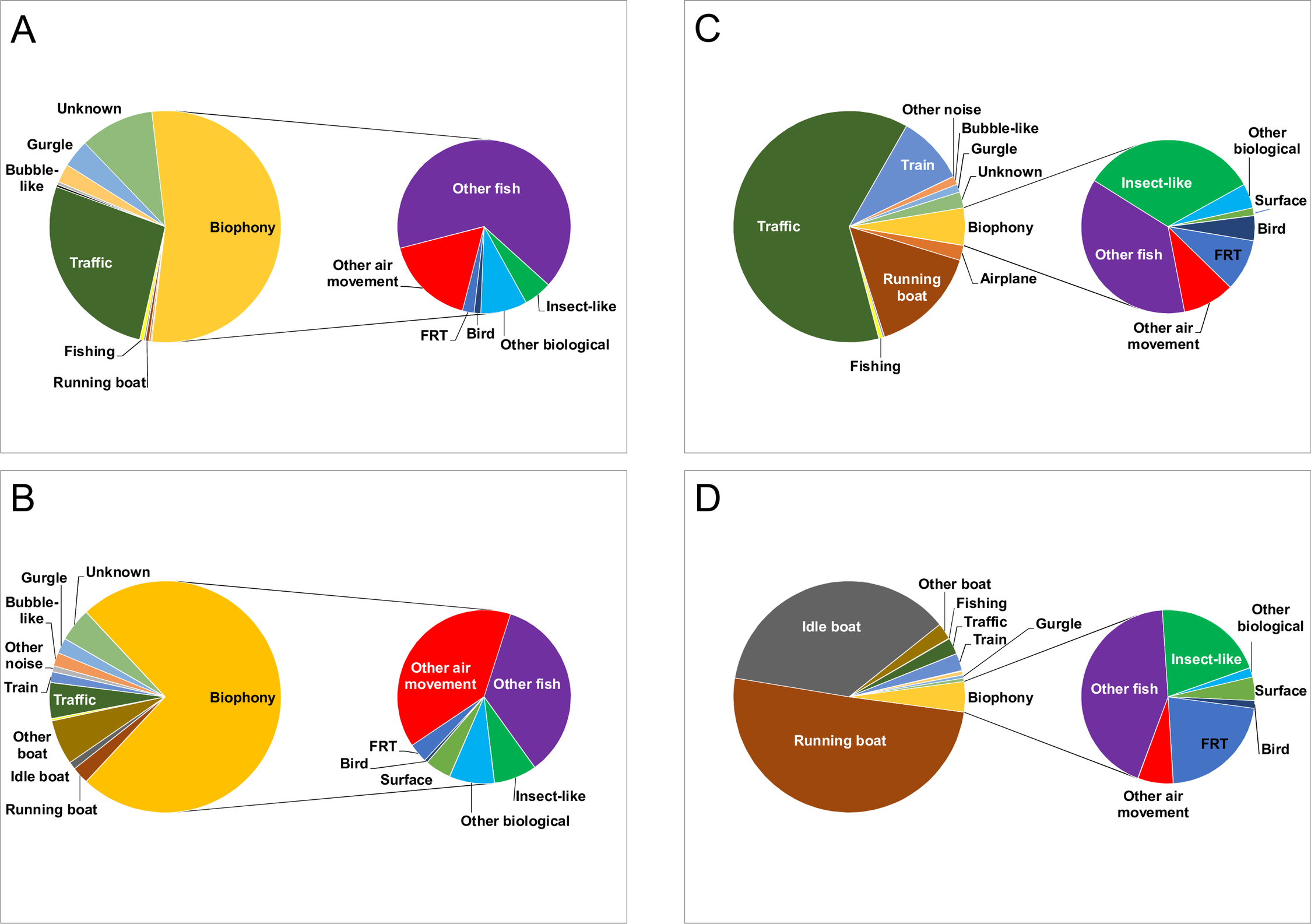

Supplement: S10 Fig — Relative contribution of anthropophony and biophony to the aquatic soundscape of non-tidal (top) and tidal (bottom) main-stem river regions during the day based on mean number of sounds per minute (A and B) mean percent time (C and D) of each sound type. Data and statistics are provided in S6 Table, while the size of the wedge represents the relative proportion of the sound out of all sounds. (TIF) [file pone.0221842.s010.tif]

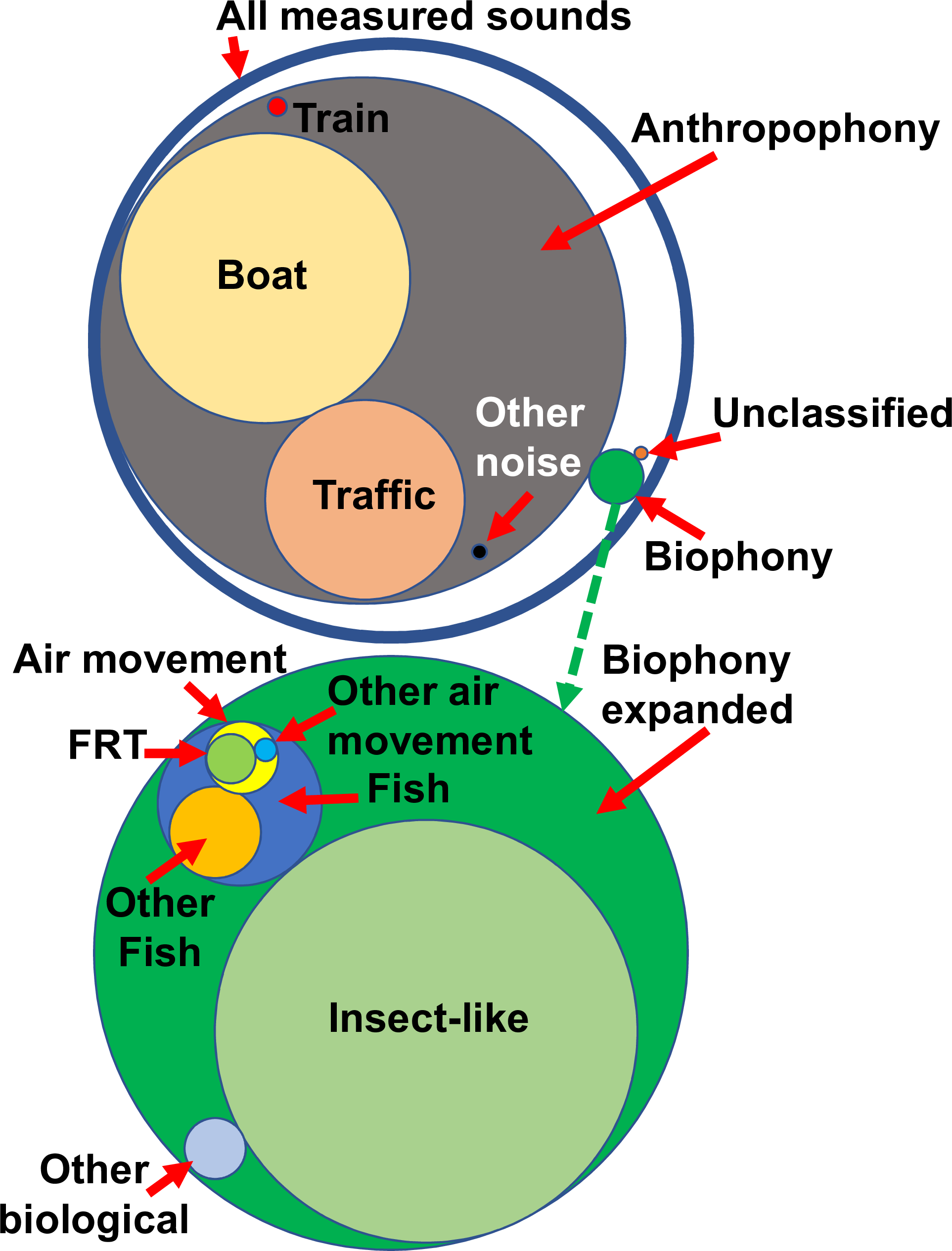

Supplement: S11 Fig — Venn diagram illustrating the relative composition of the anthropophony and biophony and their major constituents to the day-time soundscape. The diameter of each circle is proportional to the mean percent of recording time for the indicated sound category. Circles within large circles represent subcomponents of the larger category. For example, the fish category contains two nearly equal subcomponents (other fish and air movement sound). (TIF) [file pone.0221842.s011.tif]

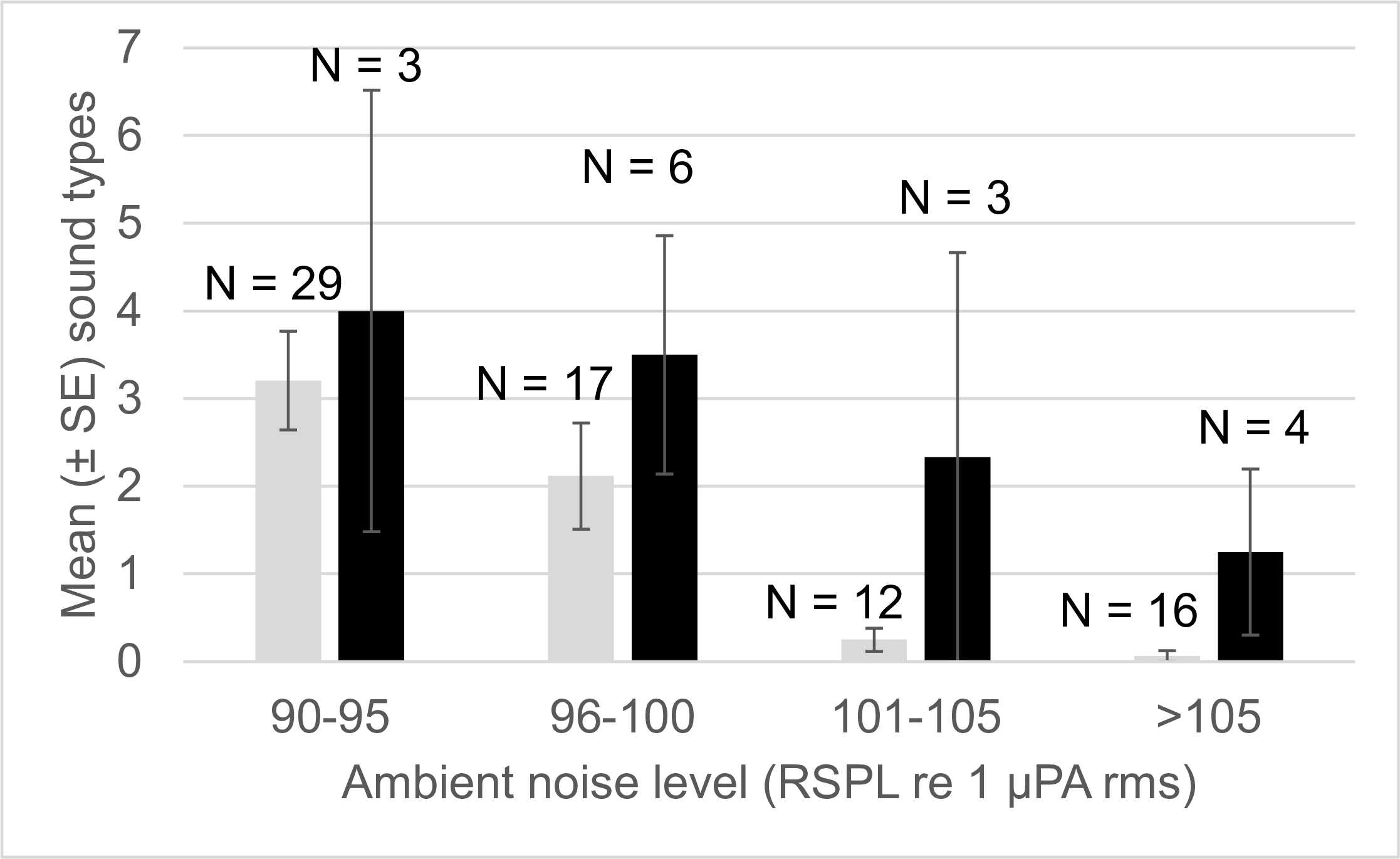

Supplement: S12 Fig — Comparison of the number of biophony sound types among locations grouped into four ambient noise levels based on received sound pressure level (RSPL). The decline in diversity form low to high noise level is highly significant (P ≤ 0.001) during the day (gray bars, N = 74), but nonsignificant during the night (black bars, N = 16). (TIF) [file pone.0221842.s012.tif]
